# ClareV: a contrastive learning framework for context-aware TRBV representations in TCR repertoires

**DOI:** 10.3389/fimmu.2026.1866480

**Published:** 2026-07-23

**Authors:** Miaozhe Huo, Yu Cheng, Tongfei Shen, Jingwan Wang, Yuepeng Jiang

**Affiliations:** 1 Department of Computer Science, City University of Hong Kong, Hong Kong, Hong Kong SAR, China; 2 Department of Electrical and Computer Engineering, National University of Singapore, Singapore, Singapore

**Keywords:** context-aware embeddings, contrastive learning, immune memory, repertoire classification, T cell receptor repertoire, TRBV

## Abstract

**Background:**

V gene segments of the T cell receptor (TCR) *β* chain (TRBV) are crucial for antigen recognition, yet previous studies have treated them as fixed categories and neglected repertoire-specific immune dynamics that inform functional diversity.

**Methods:**

We introduce ClareV, a contrastive learning framework that partitions T cell receptor repertoires into V gene-specific groups to extract data-driven embeddings capturing these dynamics. We evaluated ClareV across three TCR repertoire cohorts spanning cytomegalovirus (CMV), gastric cancer, and multi-cancer settings.

**Results:**

In the Emerson2017 CMV cohort, the ClareV Random Forest (RF) Fusion model improved AUC by 14.3% over the strongest V-family usage-only baseline (linear SVM) and by 13.3% over the strongest V-gene usage-only baseline (Random Forest). These results indicate that ClareV embeddings capture additional repertoire-level information beyond V-frequency summaries. Ablation studies showed that frequency-weighted bagging and contrastive V-bag learning both contributed to performance, outperforming simple pooling and non-contrastive learned-bag controls. In the CMV cohort, downstream embedding analyses showed that ClareV representations partially recapitulated IMGT-defined V-gene structure while retaining a CMV-responsive, non-sequence component that was largely orthogonal to classical V-usage signals.

**Discussion:**

These findings support adaptive V-gene representations as a compact feature space that complements V-frequency summaries for repertoire-level classification.

## 1 Introduction

The extraordinary diversity of T cell receptors (TCRs), generated through V(D)J recombination, underpins the adaptive immune system by enabling the recognition of a vast array of antigens (1–4). An individual’s TCR repertoire, comprising approximately 10^7^ to 10^8^ unique sequences (2, 5), not only records past immunological events but also represents a reservoir for future responses. Each TCR consists of an *α* chain and a *β* chain, each containing three complementary-determining regions (CDRs). These hypervariable regions are widely regarded as the primary determinants of the antigen specificity of the TCR (6–8). In this study, we analyze exclusively the T cell receptor *β*-chain V gene (TRBV) repertoire; accordingly, *TCR repertoire* throughout this report refers to the TRBV clonotypic repertoire.

Many studies to date have concentrated on analyzing the CDR3 region of the TCR-*β* chain, often neglecting the V gene segments (TRBVs), which encompass the CDR1, CDR2, and most portions of the framework regions (9, 10). Importantly, the TRBV segment also shapes the CDR3 itself: it contributes the germline-encoded 5^′^ portion of that loop, whereas the complete CDR3 is generated at the V-(D)-J junction and in most rearrangements includes a diversity (D) segment (1). Because IMGT annotates each rearranged sequence by its constituent TRBV and TRBJ genes (11), this germline-defined TRBV identity is a well-defined feature that should not be discarded when encoding a repertoire. This oversight may undermine the reliability of conclusions drawn exclusively from CDR3 analysis. Given emerging evidence for the role of V gene segments in immunological studies (12–14), a key open question is how to convert V-gene information into numerical representations that improve computational analyses of TCRs. In parallel, immune repertoire analysis has rapidly expanded through high-throughput sequencing and dedicated computational frameworks, highlighting the need for representations that can scale across large and heterogeneous repertoires (15–18).

Conventional analyses treat V genes as fixed and discrete categories, primarily focusing on counting the frequency of V genes across investigated cohorts (19, 20). For example, Miron et al. performed a quantitative analysis of V gene usage among memory T cell subsets to characterize the patterns of clonal expansion and inter-site sharing (21), while Dong et al. reported an association between increased TRBV201 usage and outcomes following programmed cell death protein 1 (PD-1) blockade in advanced non-small cell lung cancer (22). Similarly, several deep learning approaches encode V genes using the one-hot representations for analyzing the interaction between TCR and epitope, and disease diagnosis (23–25). However, by treating V genes as discrete categories, these methods overlook functional similarities among V gene segments and the possible associations between the V gene and other parts of the TCR, thereby limiting the models’ capacity to learn context-dependent dynamics intrinsic to the TCR repertoire.

These limitations motivate computational readouts that can capture context-dependent immune adaptation across exposure and disease contexts (17, 18). Such readouts are relevant to immune memory and disease cohort studies, where repertoire states may vary with prior antigen exposure, clonal history, and cohort background (21, 22, 26). However, existing V-gene analyses commonly represent V genes as predefined categories in usage analyses (27–30). Although useful for cohort-level comparison, this convention does not explicitly model relationships among V genes and may underrepresent repertoire-specific immune context beyond IMGT-defined labels. This gap motivates methods that can learn repertoire-adaptive V-gene structure from the sequence context within each repertoire.

To address this need, we propose the ClareV (Contrastive Learning of Adaptive Receptor Embeddings for V genes) framework. ClareV generates repertoire-adapted V gene representations that capture both inter-gene similarity and repertoire context through a V gene bagging design followed by contrastive learning. By constructing frequency-weighted V-specific bags and learning from matched repertoire context, ClareV represents each V gene as a repertoire-adaptive embedding rather than a fixed categorical label. We evaluated ClareV on three peripheral blood mononuclear cell (PBMC) TCR repertoire classification settings, including cytomegalovirus (CMV), gastric cancer, and multi-cancer cohorts, and found that the ClareV Random Forest (RF) Fusion model achieved the highest mean AUC among tested methods across cohort and resolution settings. Ablation analyses further showed that both frequency-weighted bagging and contrastive V-bag learning contributed beyond simple pooling and non-contrastive learned-bag controls. In the CMV cohort, we further examined the learned V-gene space and found that ClareV partially recapitulated IMGT-defined organization while retaining CMV-responsive embedding structure largely distinct from classical V-frequency signals, supporting complementarity between ClareV embeddings and conventional V-frequency summaries. Integrating repertoire-adapted V gene features into TCR machine learning models reduces reliance on purely categorical V representations and improves model expressiveness.

## 2 Materials and methods

### 2.1 TCR data acquisition and preprocessing

TCR repertoire sequencing data were obtained from the Emerson cohort (26), which comprises 760 individuals, including both healthy controls and CMV-infected patients. V and J gene annotations were interpreted according to the IMGT nomenclature/database standard (11).

The following filtering strategies were applied to the repertoire data (1): removal of CDR3-*β* sequences with amino acid lengths less than 10 (2), exclusion of nucleotide sequences containing stop codons, and (3) retention only of amino acid sequences initiating with cysteine (C), terminating with phenylalanine (F), and (4) removal of sequences with ambiguous V or J gene annotations. To reduce computational cost and minimize the influence of ultra-low-frequency background clonotype signal before V-bag construction, we retained the top 10,000 most abundant clonotypes from each repertoire by frequency (**Supplementary Note 1.1**; **Supplementary Figure 1**). Cohort-level distribution summaries of V-gene usage, J-gene usage, and CDR3 amino-acid length after preprocessing are provided in **Supplementary Figure 2**.

### 2.2 ClareV framework for adaptive V gene representations

**Figure 1:**
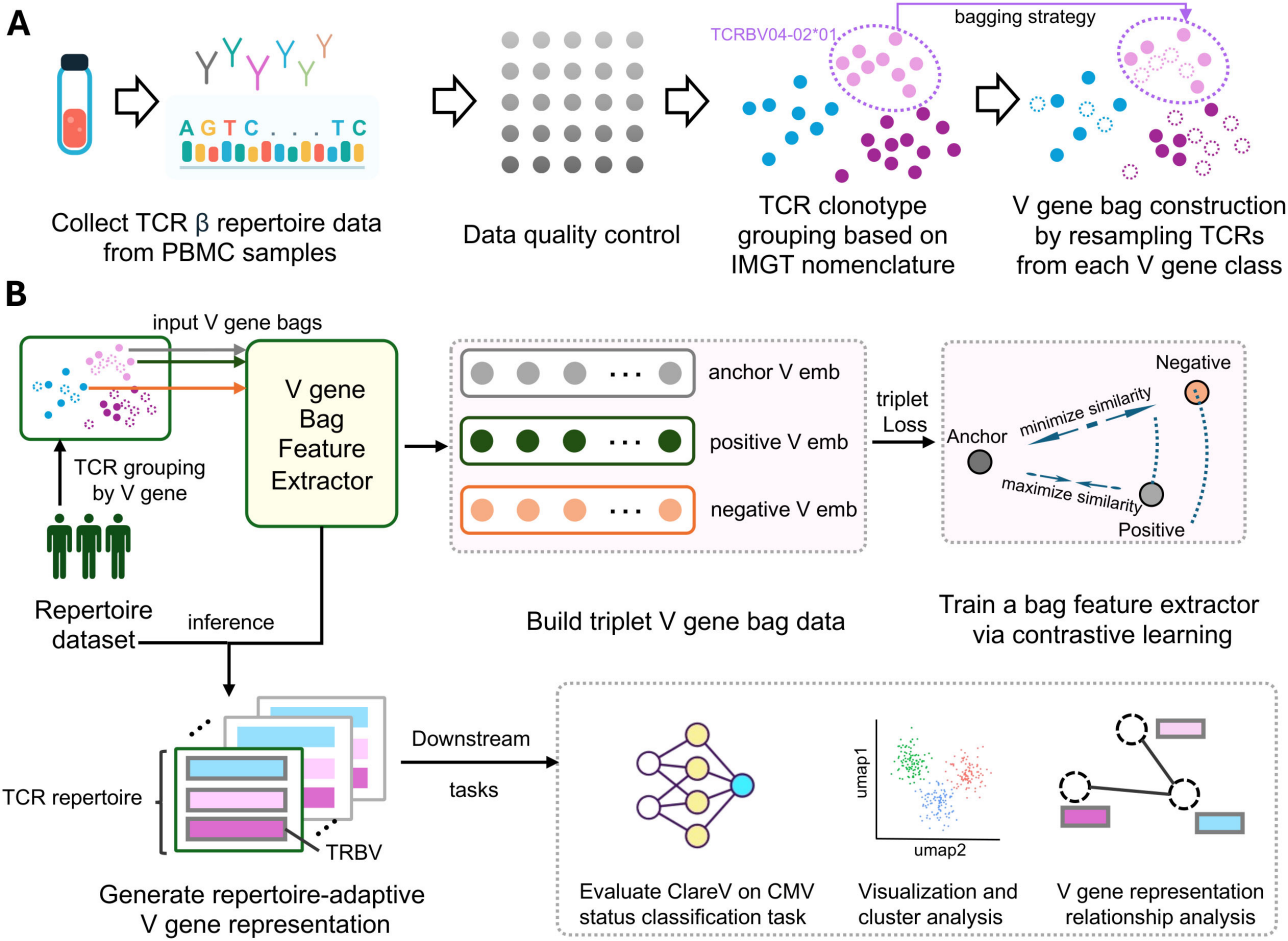
Schematic diagram of learning and applying ClareV for V gene representations. **(A)** ClareV filters the TCR sequences from the immune repertoire, assigns each TCR to the corresponding V gene type according to the IMGT nomenclature, and finally forms the V gene bags by bagging. **(B)** ClareV applies a V gene bag feature extractor to achieve repertoire-adaptive V gene embedding learning. After feature extractor training, each V gene in each repertoire is encoded into a personalized V gene embedding for downstream tasks.

The ClareV framework generates repertoire-specific representations of V genes by integrating deep learning with contrastive optimization, as illustrated in **Figure 1**. This architecture is motivated by prior observations that TCRs with identical V gene annotations can display functional redundancy while dynamically adapting to repertoire-specific immune contexts (31, 32). The proposed framework comprises three stages as detailed in the following sections.

#### 2.2.1 V gene bag construction

In this stage, TCR*β* sequences (each defined by its TRBV and TRBJ gene segments together with the intervening CDR3 region formed by V-(D)-J recombination) are first encoded into 120-dimensional vectors using the TCR2vec (33) framework, which has demonstrated its embedding efficiency in facilitating the quantification of TCR selection (34). The initial embedding vectors contain the information of CDR3 region as well as the gene segments, as TCR2vec was pre-trained on full-length TCR*β* sequences.

**Figure 2:**
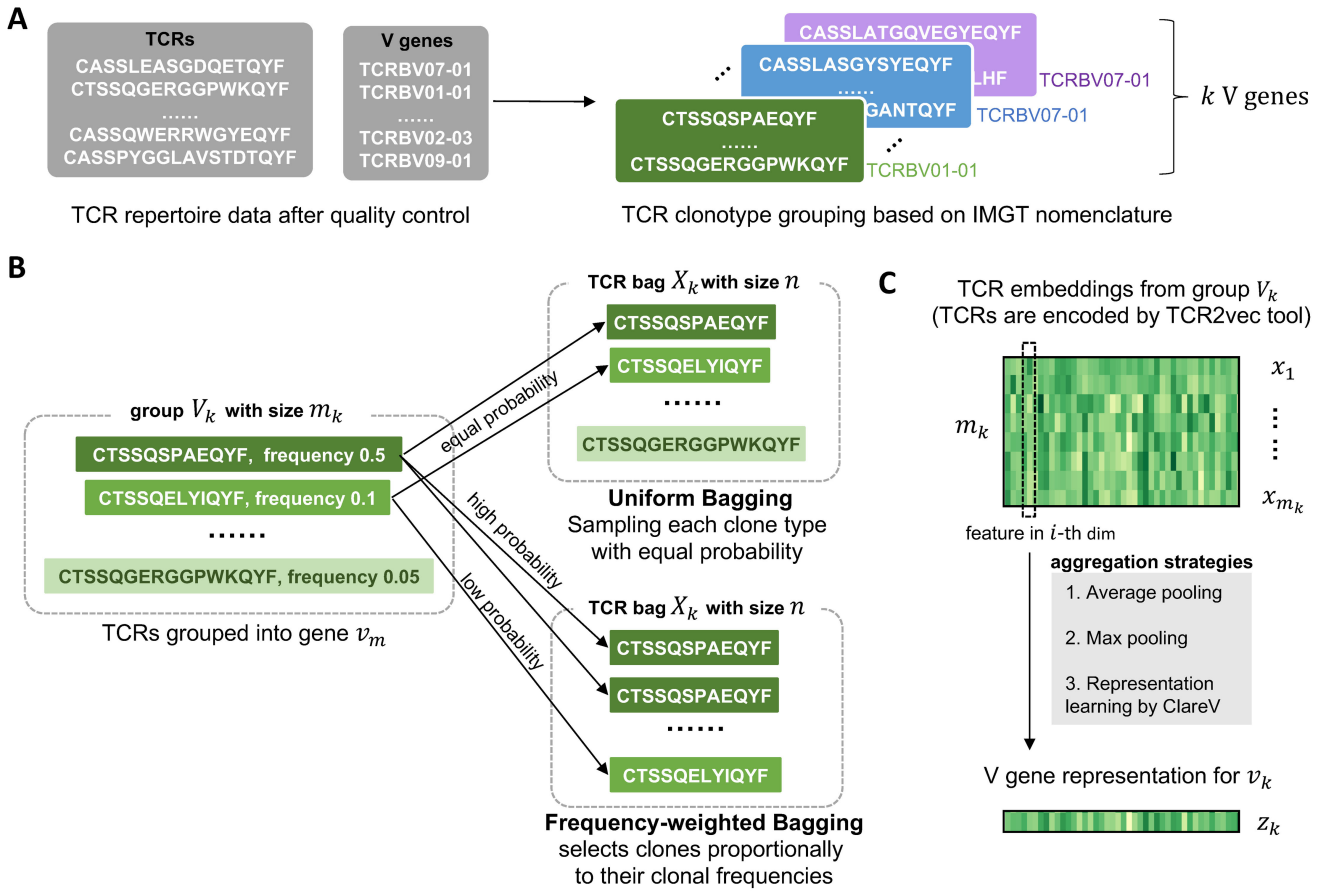
Schematic diagram of V gene-specific grouping, bagging, and V gene feature aggregation methods. **(A)** After quality control, CDR3s in the TCR repertoire are grouped according to the V gene annotation of each TCR clonotype. **(B)** For each V gene-specific group $V_{k}$ obtained in **(A)**, the ClareV framework employs two optional bagging strategies (uniform bagging and frequency-weighted bagging) to construct a V gene bag containing a fixed number n from all TCR clonotypes. **(C)** From the V gene-specific group $V_{k}$ constructed in **(A)**, the ClareV framework derives a V gene representation. Alternatively, other aggregation methods, such as average pooling and max pooling, can also be applied to obtain the embedding **z***_k_*for $V_{k}$.

These embeddings are further organized into groups $\left\{V_{1},V_{2\cdots },V_{k},\right\}$ according to their corresponding IMGT V gene annotations (**Figure 2A**) (11). To ensure a consistent V-category dimension across repertoires, only V categories present in all repertoires were retained for downstream analyses. Specifically, TCR clonotype embeddings are first grouped by V gene annotation, and each V gene-specific group is then aggregated into a fixed-size V gene bag for downstream representation learning.

As a result, for each V gene *v_k_*, the corresponding group forms an embedding with the dimension of *m_k_*× 120, where *m_k_*represents the number of clonotypes associated with *v_k_*and 120 is the embedding size. Then, a bagging strategy is applied to each group to construct organized V gene bags, each of which contains a fixed number of *n* clonotypes. Consequently, each original embedding of the V gene group (*m_k_*× 120) is converted into a bag *X* with a dimension of *n* × 120. Two distinct bagging methods were implemented to achieve this transformation, as shown in **Figure 2B**.

The first employed method, *uniform bagging*, samples TCRs from the original V gene group with equal probability (**Figure 2B**). The second utilized method, *frequency-weighted bagging*, draws TCRs with probabilities proportionally to their clonal frequencies in the repertoire (**Figure 2**), thereby preserving the clonal expansion information that is considered associated with antigen exposure (35, 36). We set frequency-weighted bagging as the default V gene bagging strategy for our downstream analyses.

#### 2.2.2 Contrastive learning for the representation of the V gene bag

Following the construction of the V gene bags, a contrastive learning approach was designed to further learn their feature representations by exploiting the similarity between the same V gene groups and their disparities in the context of different repertoires. As a result, this strategy keeps embeddings within each V gene group close while keeping bags from different repertoires in clearly distinct regions of the embedding space.

**Figure 3:**
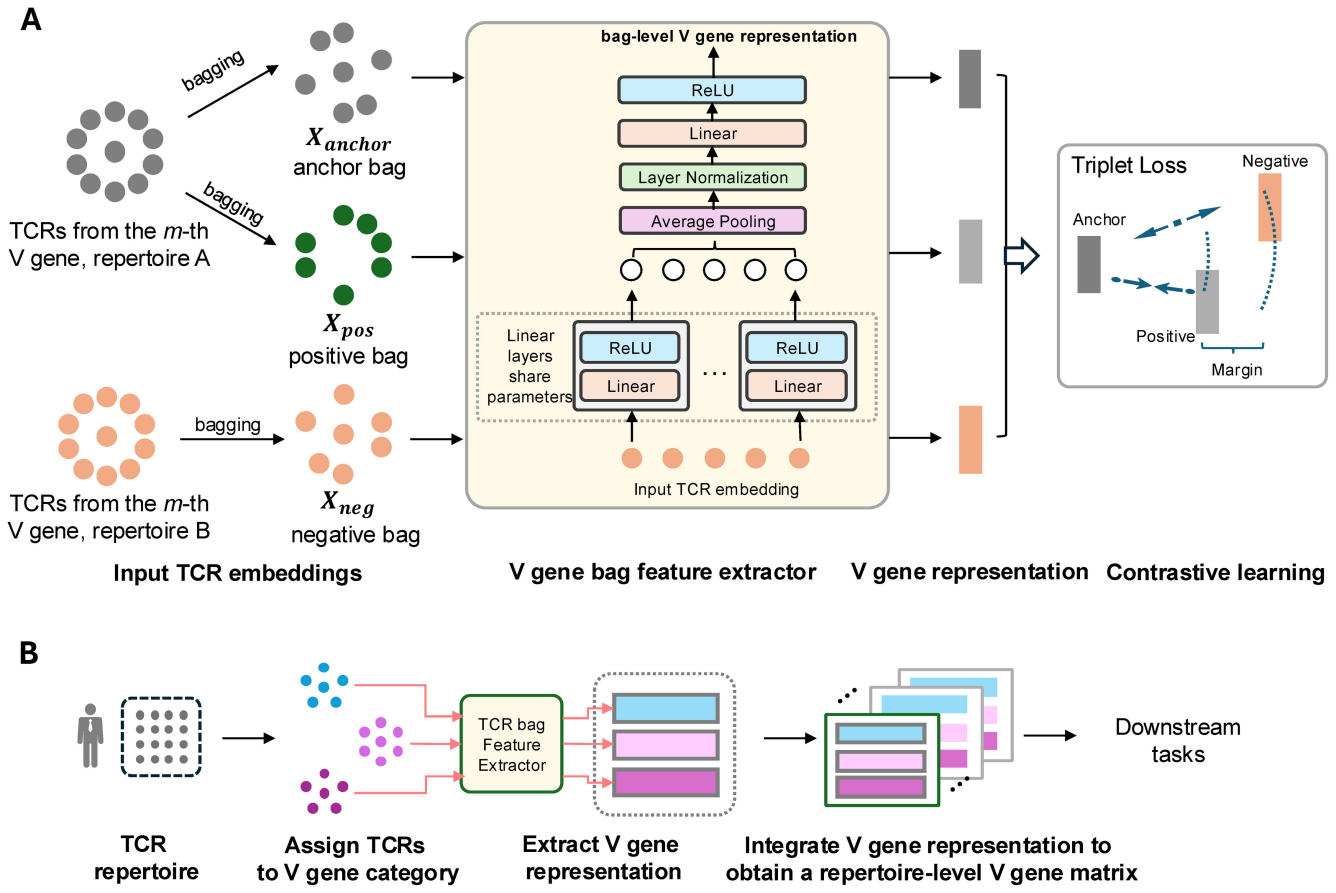
Schematic of V gene bag sampling and feature extraction in ClareV framework. **(A)** Model architecture using bagging methods and a shared-weight bag feature extractor to compute a cosine similarity-based contrastive loss. **(B)** Workflow for converting repertoire data into V gene representations for downstream analysis.

To achieve this, triplet bags $\left(X_{\text{anchor}},X_{\text{pos}},X_{\text{neg}}\right)$ is generated using the following procedures: for a given V gene and a single repertoire, we independently apply the bagging strategy twice to generate two V gene bags that serve as the anchor and positive samples respectively, ensuring that they share the same contextual background. In contrast, the negative sample is drawn from a V gene bag with the same V gene but from a different repertoire, thereby mimicking the heterogeneity across repertoires (6, 37). This sampling strategy guarantees that each triplet encapsulates both within-repertoire consistency and between-repertoire variation (**Figure 3A**).

For each triplet, a shared bag feature extractor generates representations for the anchor, positive, and negative samples. The training of this shared feature extractor is accomplished by the triplet loss, which is defined as:


$$L_{cl}=\underset{\text{triplets}}{\sum }\text{max }(-\text{ Sim}(\text{Φ}(X_{\text{anchor}}),\text{Φ}(X_{\text{pos}}))+\text{ Sim}(\text{Φ}(X_{\text{anchor}}),\text{Φ}(X_{\text{neg}}))+\gamma ,\;0)$$

where Φ(·) represents the shared bag feature extractor; $\text{Sim}(e_{i},e_{j})$ computes the cosine similarity between $e_{i}$ and $e_{j}$; γ is the margin value to control the separation between positive and negative pairs; we recommend a default of 0.1, which may be reduced for smaller cohorts. This loss function encourages the extractor to draw the anchor and positive representations closer while pushing the negative representation farther apart, so that the embedding space captures repertoire-context distinctions between V-gene groups (38–40). The well-trained V gene bag feature extractor established through this process is used for generating context-aware TRBV representations for subsequent analyses (**Figures 1B**, **3B**).

#### 2.2.3 Generation of repertoire-adapted V gene embeddings

Finally, the trained V gene bag feature extractor is employed to derive context-aware representations from the V gene bags constructed in the first stage. Specifically, each V gene bag corresponding to a particular V gene is transformed into an embedding vector. These embedding vectors are then aggregated into a comprehensive repertoire-level embedding matrix with shape *k* × 120, where *k* denotes the number of V genes in the dataset. These adaptive representations are subsequently employed for downstream tasks, including visualization analysis, IMGT V gene annotation alignment, and hierarchical clustering analysis (see **Figure 3B**). A detailed description of the feature extractor is provided in the subsequent sections.

### 2.3 Architecture of the V gene bag feature extractor

The triplet samples are processed through a shared bag feature extractor designed to capture deep bag-level representations. Initially, each clone embedding **e***_i_*is projected via a linear transformation $W_{1}\in {\mathbb{R}}^{128\times 120}$, which incorporates a dropout rate of 20% to prevent overfitting. This projection is followed by layer normalization (with *ϵ* = 10^−5^) and ReLU activation to produce stabilized intermediate features **h***_i_*. Then, these features are aggregated by mean pooling:


$$b=\frac{1}{n}\sum_{i=1}^{n}h_{i},$$

where *n* = 50 is the fixed bag size. Finally, a secondary linear projection $W_{2}\in {\mathbb{R}}^{120\times 128}$ maps the pooled vector into the target embedding space, yielding the final bag-level representation. **Figure 3A** provides an overview of this architecture.

### 2.4 Training and evaluation of the V gene bag extractor

#### 2.4.1 Data split and fold-wise extractor isolation

For repertoire classification experiments, we predefined repertoire-level nested 5-fold splits. For each fold, the 20% hold-out test repertoires were excluded from both extractor training and classifier training, and the remaining 80% development repertoires were further split at the repertoire level into a 9:1 training/validation subset. A fold-specific extractor was trained on this development data, and the checkpoint with the lowest validation contrastive loss $L_{\text{cl}}$ was used to generate fold-matched ClareV features for downstream classification in the same fold. Outer-test repertoires never participated in extractor training, validation, or downstream classifier fitting within their own fold’s pipeline.

For descriptive embedding-space analyses on the Emerson2017 cohort, we additionally trained a separate Emerson analysis V-gene bag extractor using an 80%/10%/10% repertoire-level split. This analysis-only extractor does not contribute to any of the reported classification AUCs.

#### 2.4.2 Training hyperparameters

All contrastive extractors were trained with Adam (initial learning rate 10^−4^, batch size 32). For Emerson2017, we used triplet margin *γ* = 0.1 with up to 40 training epochs; for the two smaller validation cohorts (Wang2022 and Hamm2020), we used a more conservative margin *γ* = 0.08 and capped training at 20 epochs to limit overfitting on the smaller triplet pool. Sensitivity of these choices to V-bag size and triplet margin is examined in **Supplementary Notes 1.1** and **1.2** (**Supplementary Figures 3**, **S4**, **S5**).

### 2.5 Assess ClareV framework on repertoire classification tasks

#### 2.5.1 Classification tasks and ClareV-based classifiers

We evaluated ClareV on repertoire-level classification tasks across the Emerson2017 CMV cohort (26) and two external PBMC TRBV repertoire cohorts, Wang2022 (28) and Hamm2020, representing gastric cancer and multi-cancer settings. Cohort definitions, label settings, and small-cohort extractor adjustments are described in **Supplementary Note 1.3**. For each repertoire, TCR clonotypes were grouped into V gene bags. Each bag was then passed through the fold-matched V gene bag feature extractor to produce a fixed 120-dimensional embedding for each V category, and these embeddings were aggregated into a repertoire-level representation (**Figure 3B**; Methods Section 2.2).

At gene resolution, we used IMGT gene-level annotations; at family resolution, we grouped genes into broader IMGT-defined families. In parallel, we computed normalized V-frequency features by counting unique TCR clonotypes per V category within each repertoire. Unless otherwise specified, TRBV representation refers to ClareV-derived V-region features at the analysis-specific resolution.

We evaluated two ClareV-based classifiers: a ClareV embedding-only classifier and a Random Forest (RF) Fusion model that combines ClareV embeddings with the output of a V-frequency Random Forest classifier. The ClareV-based classifiers were implemented in PyTorch (torch=1.9.0). All repertoire classification models used the same predefined nested 5-fold repertoire splits, and each outer fold used the corresponding fold-matched extractor from Methods Section 2.4. We used AUC as the primary evaluation metric and reported ACC and F1 as secondary summaries; detailed classifier settings and metric definitions are provided in **Supplementary Notes 1.4** and **1.5**.

For paired superiority analyses, we compared ClareV RF Fusion with reference baselines on matched outer-fold predictions. Paired AUC differences were estimated with 95% confidence intervals and one-sided *P*-values using a fold-stratified paired bootstrap (*B* = 5000), resampling identical patient indices for the two compared methods within each outer fold. Complete paired AUC differences are reported in **Supplementary Table 4**.

#### 2.5.2 Baseline and ablation models

We compared ClareV-based classifiers with usage-only repertoire classifiers trained on normalized Vfrequency features, including Logistic Regression, Random Forest, SVM with linear and RBF kernels, CNN, and MLP baselines. For representation-level ablation, we also evaluated two non-learned pooling strategies, average pooling and max pooling, applied to each V gene group $V_{k}$. To isolate the contribution of contrastive learning, we further introduced two non-contrastive learned V-bag baselines using the same V-bag inputs as ClareV: a supervised V-bag encoder and an Attention MIL V-bag encoder. Implementation details for the usage-only classifiers and non-contrastive learned V-bag baselines are provided in **Supplementary Notes 1.4** and **1.6**.

### 2.6 V gene embedding visualization by UMAP method

For UMAP visualization, annotation-alignment analysis, and hierarchy analysis on the Emerson2017 cohort, we used the Emerson analysis V-gene bag extractor described in Methods Section 2.4. We then encoded V gene bags from each repertoire to generate repertoire-adaptive V gene embeddings for all V genes. Similarly, we employed average pooling and max pooling as baseline methods to compare against the performance of the ClareV V gene representation learning approach.

The embeddings derived from multiple repertoires were then pooled together and subjected to dimensionality reduction via UMAP (41), using the parameters ‘n_components=2’, ‘n_neighbors=100’, ‘min_dist=0.2’, and the ‘cosine’ metric. A scatter plot was generated in which each point corresponds to the two-dimensional UMAP projection of a 120-dimensional V gene embedding from a given repertoire. These visualizations were further colored based on the annotations of the IMGT V gene or patient identifiers associated with the repertoires, thereby facilitating the intuitive exploration of the V gene embedding data distribution.

### 2.7 Evaluation of V annotation alignment in embedding space

We first quantified gene-level annotation alignment by evaluating how well k-means clusters of learned V embeddings matched IMGT V gene labels. We used four clustering-based metrics: one minus normalized mutual information (1-NMI), one minus adjusted Rand index (1-ARI), one minus purity (1-Purity) (42), and the Cluster Entropy Measure (43). Lower values indicate closer agreement between clustering outcomes and IMGT labels. For each embedding method, k-means was run with cluster numbers from 10 to 100 using scikit-learn (44). Detailed metric definitions are provided in **Supplementary Note 1.7**.

We then evaluated family-level organization in the same embedding space by re-annotating gene-level embeddings with IMGT V family labels, visualizing them with UMAP, and quantifying separation with silhouette coefficients (45). For families containing multiple genes (TCRBV04, TCRBV05, TCRBV06, TCRBV07, TCRBV08, TCRBV10, TCRBV11, and TCRBV12), we overlaid family centroids to summarize cluster centers. Calculation details are provided in **Supplementary Note 1.8**.

### 2.8 Hierarchical clustering analysis of ClareV-learned TRBV embeddings

Using the ClareV TRBV embeddings obtained from the Emerson dataset, we performed hierarchical clustering to characterize embedding-space organization among these repertoire-adapted embeddings. For each V gene, a population-level embedding was computed by averaging the repertoire-level TRBV embeddings $z^{ClareV}$, as:


$${\bar{z}}_{k}=\frac{1}{N}\overset{N}{\underset{n=1}{\sum }}z_{kn}^{ClareV}$$

where *k* indexes the V gene category and *n* indexes the individual repertoire for the *k*-th gene, with *N* being the number of repertoire in the whole dataset. This process produced a matrix $\bar{z}\in {\mathbb{R}}^{K\times d}$, where *K* denotes the number of unique V genes and *d* represents the dimensionality of each embedding, here *d* = 120. Hierarchical clustering was then performed on $\bar{z}$ using the cosine distance as the similarity measure, generating a dendrogram that delineates embedding-space organization among V genes. Additional computational details are provided in **Supplementary Note 1.9**.

### 2.9 V-embedding geometry and CMV-associated shift analyses

To further interpret the Emerson2017 V-gene embedding space, we used the Emerson analysis V-gene bag extractor described in Methods Section 2.4. We computed repertoire-specific ClareV embeddings for retained V genes and summarized each V gene by its centroid across repertoires. We then compared ClareV V-gene geometry with IMGT amino-acid sequence similarity, tested whether genes from the same IMGT family were closer than genes from different families, and compared these patterns with raw TCR2vec centroid baselines.

We also assessed whether individual V genes showed CMV-associated embedding shifts in ClareV space. For each V gene, embeddings were partitioned by Emerson2017 CMV status, and the CMV-positive and CMV-negative centroids were compared. We evaluated per-V shift magnitudes, compared the embedding shift ranking with classical V-frequency association statistics, and tested whether per-V shift directions were jointly structured under a CMV-label permutation null. Detailed statistical procedures are provided in **Supplementary Notes 1.10** and **1.11**.

## 3 Results

### 3.1 ClareV features facilitates the repertoire classification task

As a case study, we evaluated ClareV on the Emerson CMV cohort (26) at two levels of V gene resolution. At the gene level, TRBV sequences were grouped based on individual TRBV gene annotations according to the IMGT nomenclature, whereas at the family level, groups were defined based on the V gene family category. Both experiments adhered to the procedures detailed in Methods Section 2.2 and employed the training methods and parameters described in Methods Section 2.4 for the V gene bag feature extractor.

Under the fold-wise hold-out protocol, extractor validation losses remained low at both resolutions: $L_{\text{cl}}\;$= 0.0074 ± 0.0004 at V-family level and $L_{\text{cl}}$ = 0.0062 ± 0.0004 at V-gene level (mean ± SD across outer folds). Using the same outer-fold splits, the fold-matched extractors were then used to generate repertoire-adaptive V embeddings for the CMV status classification task. Detailed methodology is provided in Methods Section 2.5.

**Figure 4:**
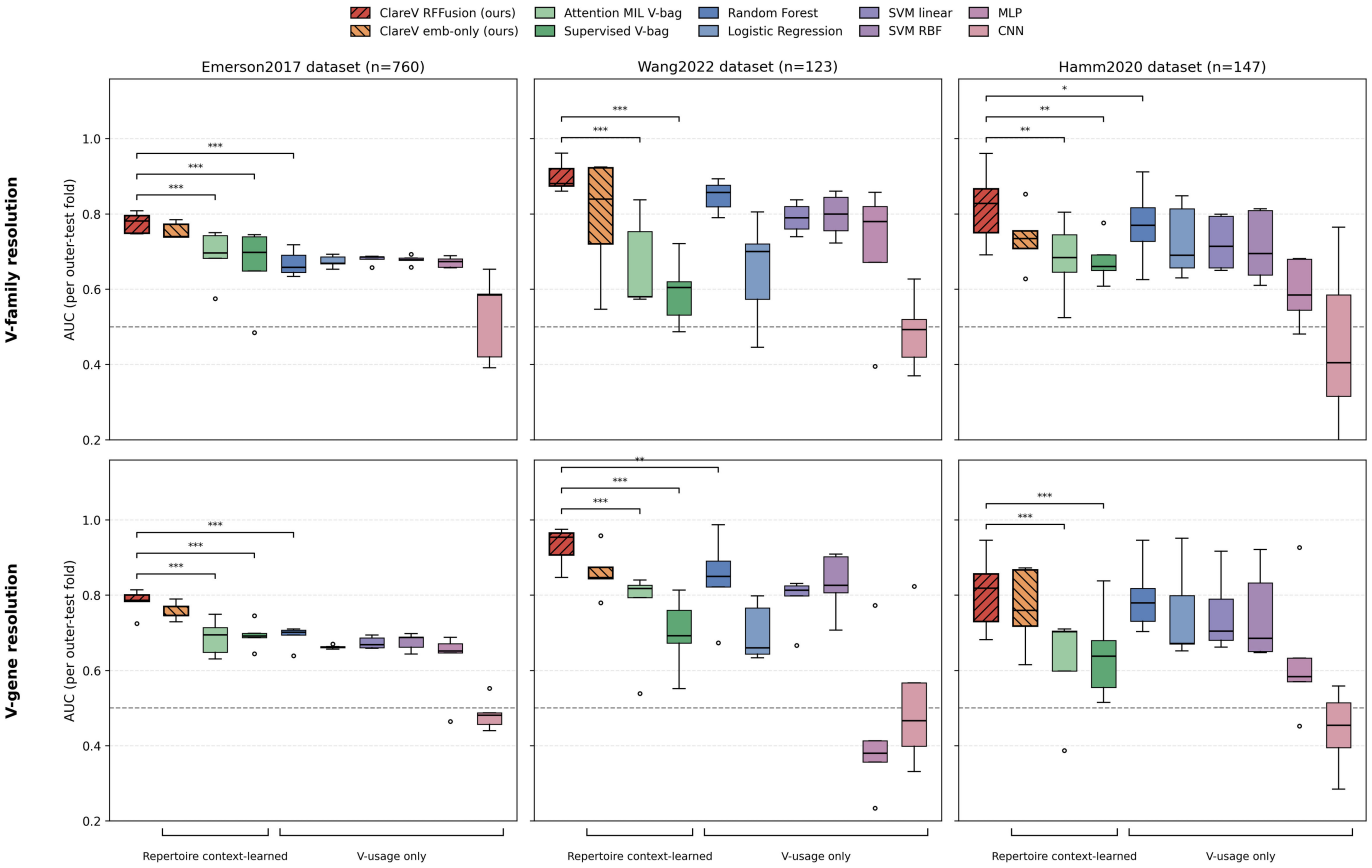
ClareV RF Fusion achieves the highest mean AUC across three repertoire cohorts at both V-family and V-gene resolution. Each panel shows nested 5-fold cross-validation AUC distributions on one cohort: Emerson2017 (left), Wang2022 (middle), and Hamm2020 (right). The top row reports V-family resolution; the bottom row reports V-gene resolution. ClareV-based methods are shown with hatched bars, and the dashed horizontal line marks chance level (AUC = 0.5). Asterisks mark one-sided fold-stratified paired bootstrap comparisons (*B* = 5000) of RF Fusion versus Random Forest V-usage or non-contrastive learned V-bag baselines: *p < 0.05, **p < 0.01, ***p < 0.001. Source data and paired tests are provided in **Supplementary Tables 1**–**S4**.

On the Emerson2017 cohort at V-family resolution, ClareV embeddings alone, classified with an MLP without V-family frequency features, achieved AUC = 0.755 ± 0.022 in nested 5-fold cross-validation (95% CI 0.721–0.788; **Figure 4**). This exceeded the highest-AUC V-family usage-only baseline, linear SVM (AUC = 0.679 ± 0.012, 95% CI 0.640–0.717). Integrating ClareV embeddings with V-family usage in the RF Fusion model further improved performance to AUC = 0.776 ± 0.028 (95% CI 0.742–0.809), a 14.3% improvement over the strongest usage-only baseline. Paired bootstrap analyses confirmed significant superiority over the Random Forest V-usage reference and over both non-contrastive learned-bag baselines (**Supplementary Table 4**).

A similar pattern was observed at V-gene resolution on Emerson2017. ClareV embedding-only classification reached AUC = 0.756 ± 0.024 (95% CI 0.722–0.788), outperforming the strongest Vgene usage-only baseline, Random Forest (AUC = 0.690 ± 0.029, 95% CI 0.652–0.727). The RF Fusion model again provided the best performance, reaching AUC = 0.782 ± 0.034 (95% CI 0.749–0.813), a 13.3% improvement over Random Forest usage. Paired bootstrap tests confirmed significant improvements over the Random Forest V-usage reference and over both non-contrastive learned-bag controls (**Supplementary Table 4**). Per-method point estimates are reported in **Supplementary Tables 1**, **S2**.

Across the two external PBMC TRBV repertoire cohorts, Wang2022 (gastric cancer vs control) and Hamm2020 (multi-cancer vs healthy), ClareV RF Fusion achieved the highest mean AUC at both V-family and V-gene resolutions (**Figure 4**; **Supplementary Table 3**). ClareV embedding-only classification did not surpass the strongest V-usage model in every external cohort/resolution setting, indicating that V-frequency summaries can remain highly informative in some cohorts. Nevertheless, integrating ClareV embeddings with V-usage features provided additional signal in the external cohorts, with the clearest gain observed for Wang2022 at V-gene resolution, where RF Fusion improved AUC by 10.1% relative to Random Forest usage alone. These results support that ClareV embeddings can complement V-frequency summaries, while the magnitude of the added benefit depends on cohort context and resolution.

### 3.2 Component contribution: feature aggregation, bagging, and contrastive learning

We next used ablation experiments on the Emerson2017 V-family classification setting to examine how three design choices contributed to ClareV performance: feature aggregation, V-bag sampling, and contrastive V-bag learning (Methods Section 2.5.2; **Table 1**).

**Table 1:** Impact of representation strategy on CMV status classification at V family level.

Feature type	Method	AUC
Simple aggregation	MLP (aggregated TRBV embedding, max pooling)	0.602 ± 0.101
	MLP (aggregated TRBV embedding, average pooling)	0.593 ± 0.040
Context-learned (non-CL)	Supervised V-bag encoder	0.663 ± 0.107
	Attention MIL V-bag encoder	0.689 ± 0.070
ClareV encoding (with CL)	MLP (ClareV TRBV embedding; uniform bagging)	0.694 ± 0.021
	MLP (ClareV TRBV embedding; frequency-weighted bagging)	0.755 ± 0.022
	RF Fusion model (ClareV TRBV embedding + V-family usage; uniform bagging)	0.671 ± 0.026
	RF Fusion model (ClareV TRBV embedding + V-family usage; frequency-weighted bagging)	**0.776 ± 0.028**

non-CL models learn V-bag representations from repertoire context but do not use a contrastive objective. ClareV rows use contrastive learning with either uniform or frequency-weighted bagging. Bold values indicate the best performance.

Simple aggregation of clonotype embeddings provided only limited classification signal. Max pooling and average pooling reached AUCs of 0.602 ± 0.101 and 0.593 ± 0.040, respectively, suggesting that fixed pooling over V-specific clonotype groups does not fully capture the repertoire-level information needed for the classification task.

Within ClareV, the V-bag sampling strategy had a clear effect. Frequency-weighted bagging outperformed uniform bagging for both embedding-only models (0.755 ± 0.022 vs 0.694 ± 0.021) and RF Fusion models (0.776 ± 0.028 vs 0.671 ± 0.026). This pattern supports the use of clonotype-frequency information during bag construction, consistent with prior evidence that clonally expanded TCRs can reflect antigen exposure-related repertoire signal (26, 35).

Learned V-bag encoders without a contrastive objective also captured useful classification signal, with the supervised V-bag encoder and Attention MIL V-bag encoder reaching AUCs of 0.663 ± 0.107 and 0.689 ± 0.070, respectively. However, ClareV with frequency-weighted bagging achieved higher AUC as an embedding-only model (0.755 ± 0.022). These results suggest that context learning within V bags is informative, while the contrastive objective generally provides a stronger representation than non-contrastive learned aggregation alone.

### 3.3 ClareV TRBV embeddings reveal repertoire-specific differentiation in the CMV cohort

We further compare repertoire-adaptive TRBV embeddings produced by the ClareV framework with those derived from simple aggregation methods in a gene-level CMV status classification context using Emerson2017 repertoires (average and max pooling; refer to Methods Section 2.5.2). Our objective was to examine whether the adaptive, contrastive learning-based approach could capture dynamic, repertoire-specific signals that extend beyond the static boundaries defined by IMGT annotations. In this evaluation, we obtained gene-level TRBV embeddings for each repertoire using the trained V gene bag feature extractor and assessed them via UMAP visualization and clustering experiments (refer to Methods Section 2.6 and Methods Section 2.7).

**Figure 5:**
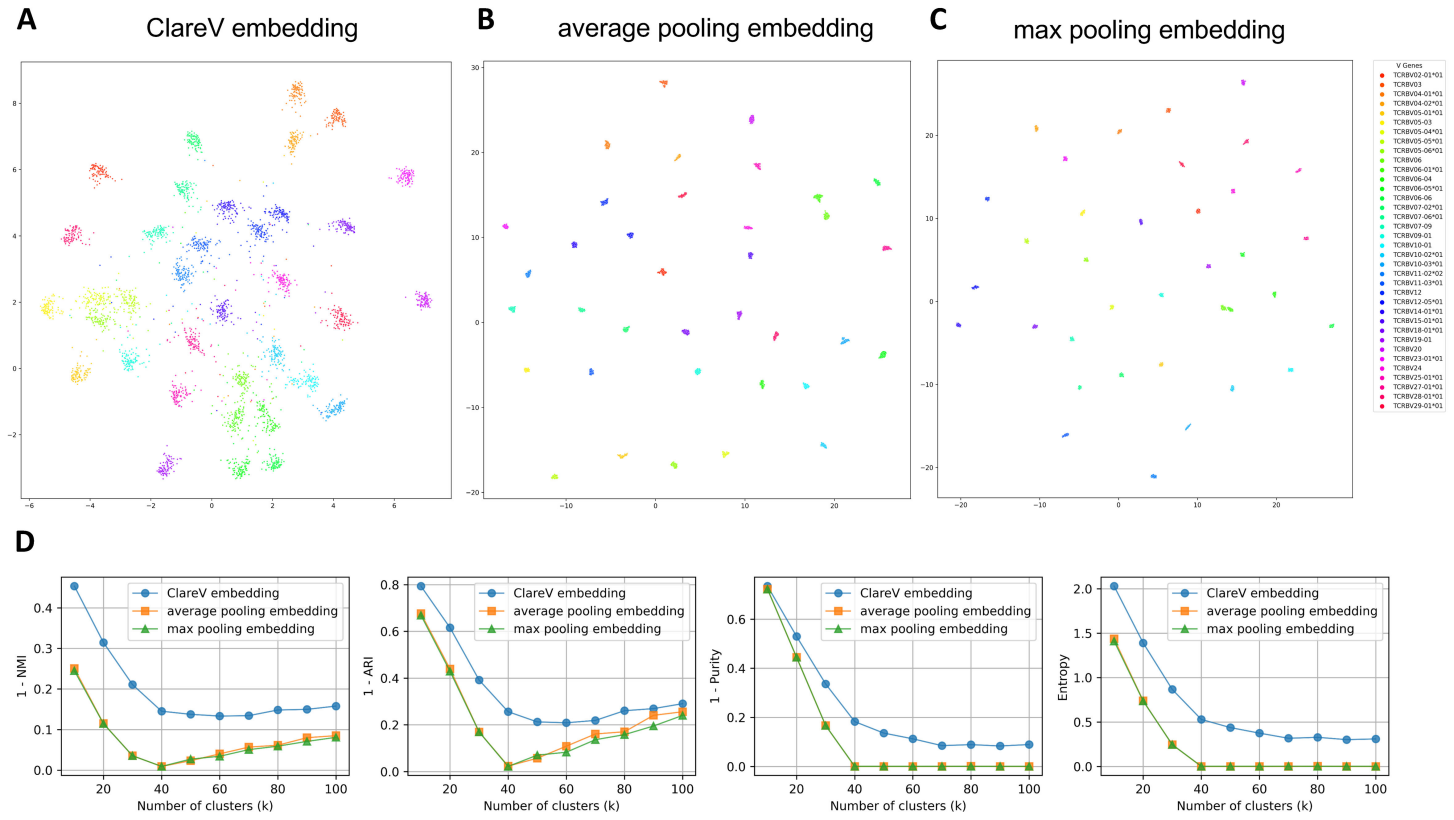
UMAP visualization of V gene embeddings. UMAP projections for three embedding aggregation strategies applied to 100 randomly selected Emerson repertoires. **(A–C)** show embeddings colored by IMGT V gene categories for ClareV, average pooling, and max pooling, respectively. **(D)** compares annotation alignment metrics (1-NMI, 1-ARI, 1-Purity, Cluster Entropy Measure) across cluster numbers. UMAP visualizations colored by CMV status are provided in **Supplementary Figure 8**.

The UMAP projections reveal that ClareV embeddings are considerably more dispersed than those produced by average and max pooling (**Figures 5A–C**). This broader distribution, with several V gene embeddings migrating across conventional IMGT clusters, indicates the capture of additional repertoire-specific heterogeneity. When colored by CMV status (**Supplementary Figure 8**), ClareV embeddings show more heterogeneous local neighborhoods than pooling baselines, which is qualitatively consistent with the quantitative performance differences in **Table 1**. In clustering analyses, we further evaluated the correspondence between the k-means clustering results of the embeddings and the IMGT classification using four metrics (1-NMI, 1-ARI, 1-Purity, and the Cluster Entropy Measure; see **Supplementary Note 1.7** for metric definitions). As shown in **Figure 5D**, the results consistently demonstrate that the ClareV framework produces embeddings that are less aligned with the static IMGT-defined boundaries than those produced by simple pooling methods, consistent with the embeddings capturing repertoire-context structure beyond IMGT category labels.

### 3.4 Reassessing V gene and family organization in the CMV cohort with ClareV-learned embeddings

Using Emerson2017 CMV repertoires and the Emerson analysis V-gene bag extractor, we annotated the repertoire-adaptive gene-level V embeddings with IMGT V family labels. These embeddings were visualized using UMAP, and their grouping consistency with the manually assigned IMGT V family labels was quantitatively evaluated using silhouette coefficients and hierarchical clustering. The goal was to evaluate whether the data-derived organization learned from repertoire data is consistent with, or differs from, the V-gene grouping defined by the IMGT nomenclature.

**Figure 6:**
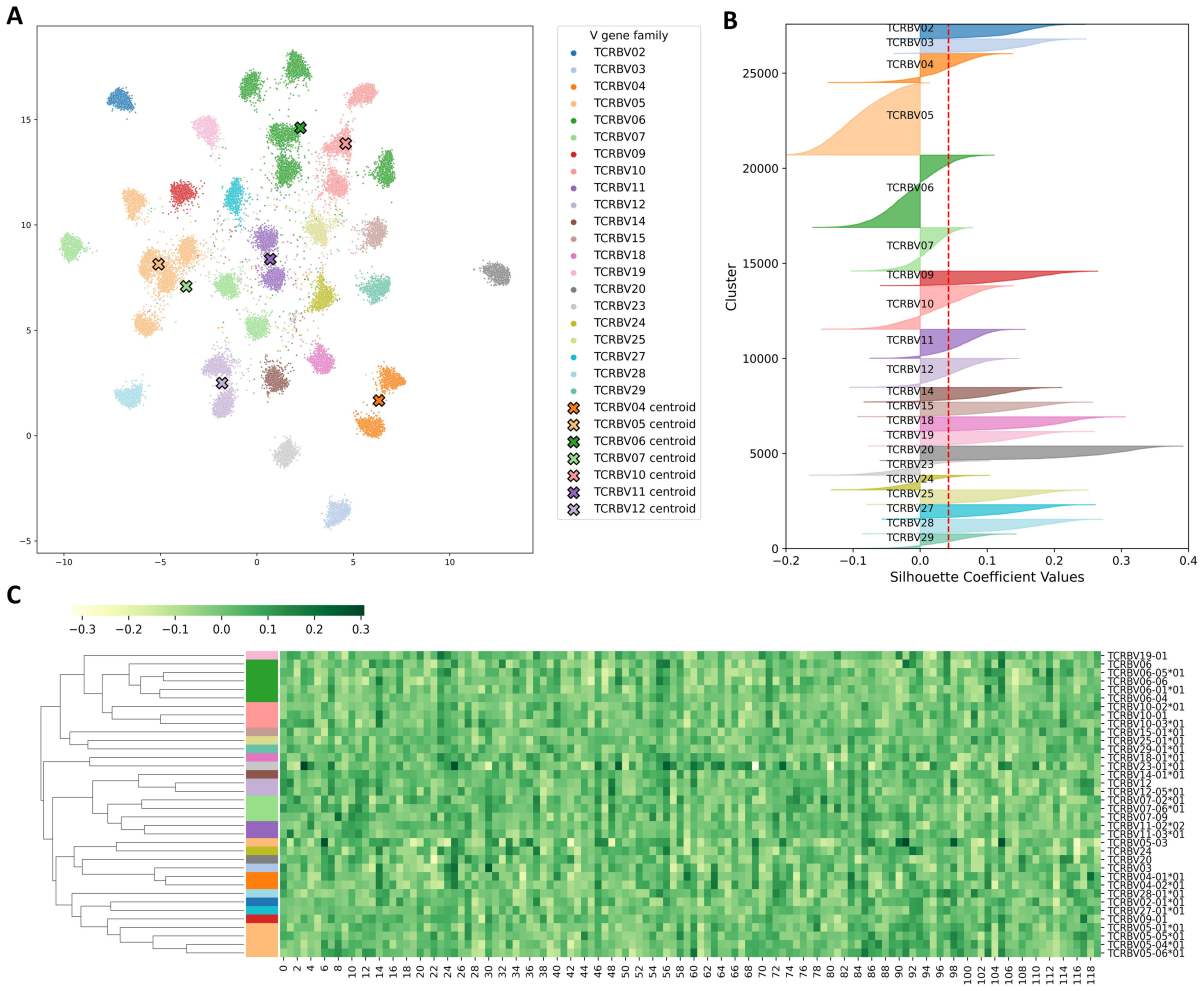
Analysis of V gene embedding space with IMGT annotation. **(A)** UMAP projection of V gene embeddings re-annotated with IMGT V family labels, with centroids marked for families having multiple genes. **(B)** Silhouette coefficient analysis of the V gene embeddings based on their re-assigned V family labels. Higher silhouette scores indicate more distinct cluster boundaries, thereby reflecting the preservation of the original V family structural organization in the embedding space. **(C)** Cosine similarity heatmap with a dendrogram illustrates inter-embedding relationships and correspondence with V family annotations.

UMAP visualization (**Figure 6A**) demonstrates that while embeddings corresponding to the same IMGTV family generally tend to form contiguous clusters, the boundaries between families are less distinct.

In the upper-central region, partial overlap among neighboring families (e.g., TCRBV06 and TCRBV10 neighborhoods) suggests inter-family proximity beyond strict IMGT boundaries. Silhouette coefficients analysis (45) further quantified these observations. Silhouette coefficients are defined on a scale from -1 to 1, with values closer to 1 indicating that clusters are highly distinct and compact (calculation details are provided in **Supplementary Note 1.8**). An overall average silhouette coefficient of 0.04224 (**Figure 6B**) indicates that the IMGT V family groups exhibit relatively low internal cohesion (source data are listed in **Supplementary Table 5**). We also observed that the scores varied across V gene family clusters. For example, TCRBV20 achieved a high score (0.2607), while TCRBV18 (0.1851) and TCRBV28 (0.1545) showed moderate cohesion. In contrast, clusters such as TCRBV05 (-0.0977), TCRBV23 (-0.0171), and TCRBV06 (-0.0148) yielded negative values, suggesting that embeddings in these groups are, on average, closer to neighboring families than to their own. Similarly, the near-zero silhouette value for TCRBV07 (0.0118) is consistent with the UMAP visualization, which shows locally indistinct boundaries with nearby families (**Figure 6B**).

Furthermore, we averaged the ClareV-learned embeddings across the Emerson cohort to generate population-level TRBV embeddings and performed the hierarchical clustering on these embeddings (Methods Section 2.8 and **Supplementary Note 1.9**). The resulting dendrogram shows that although V genes from the same IMGT family tend to cluster together, additional embedding-space groupings are observed (**Figure 6C**). For instance, TCRBV04 and TCRBV03 converge at a common internal node, and TCRBV11 remains closely aligned with TCRBV07-related branches. In addition, TCRBV02 and TCRBV27 appear closely grouped, while TCRBV19 aligns with a TCRBV06-related branch, indicating embedding-space groupings not fully aligned with the IMGT hierarchy. The observed pattern is consistent with prior CMV repertoire studies showing strong antigen-driven selection in V/J organization. In HLA-B*44:03-restricted CMV responses, high-throughput sequencing showed highly skewed, restricted V/J usage and extensive shared public clonotypes (46). In CMV-specific cytotoxic T-cell repertoires after allo-SCT, biased TRBVTRBJ usage and recurrent shared TCR patterns were also observed across patients (47). Together, these studies support the interpretation that IMGT provides a general reference taxonomy, while context-specific immune selection can reshape repertoire organization in real data.

These results show that ClareV’s data-driven, repertoire-adaptive embeddings recapitulate the broad organization of IMGT V-gene families while also exhibiting embedding-space proximities that do not strictly follow the IMGT hierarchy. We interpret these proximities as data-derived organizational patterns observed in the Emerson2017 CMV cohort that may motivate further hypothesis generation.

### 3.5 CMV-associated V-embedding geometry beyond V usage

The classification gains reported in Section 3.1 indicate that the ClareV embedding contributes information beyond classical V-usage frequencies, but they do not directly characterize what kind of structure the embedding learns. We therefore examined the geometry of the ClareV V-gene embedding on the Emerson2017 CMV cohort using the Emerson analysis V-gene bag extractor. Statistical details are provided in **Supplementary Notes 1.10** and **1.11**.

We first assessed whether ClareV’s data-driven V representation respects the IMGT V-gene taxonomy, which is defined from amino-acid sequence similarity and therefore provides a sequence-based reference. The Mantel test (48) between pairwise V-V cosine distance in the ClareV embedding and pairwise IMGT amino-acid identity showed a significant correlation (Spearman *ρ* = 0.43, *P*_perm_ *<* 10^−3^; **Supplementary Figure 9B**). At the V-family level, V genes from the same IMGT family were closer to each other in the embedding than to V genes from other families (**Supplementary Figure 9A**). These results indicate that the contrastive embedding retains sequence-grounded V-family structure without explicit family-level supervision.

At the same time, the ClareV geometry was not a simple sequence echo. The same Mantel comparison applied to TCR2vec baseline V-V centroid distances produced a much stronger sequence correlation (*ρ* = 0.90), whereas ClareV showed a more moderate relationship with sequence identity. This pattern suggests that ClareV preserves part of the IMGT-related V-gene organization while also reorganizing the embedding according to repertoire-context information learned during contrastive training.

We then asked whether this repertoire-context component was related to CMV status. We compared classical per-V usage association statistics, a common analysis strategy in CMV repertoire studies (26, 49, 50), with CMV+/− centroid shifts in the ClareV embedding. V-usage associations were strong in this cohort, but the ClareV-derived per-V shift ranking was essentially uncorrelated with the V-usage ranking (Spearman *ρ* = +0.00, *P* = 0.99; **Supplementary Figure 9C**). The embedding shifts were nonetheless jointly non-random under a CMV-label permutation test (*P*_perm_ *<* 10^−3^; **Supplementary Note 1.11**).

Together, these analyses support a restrained interpretation: in the Emerson2017 CMV cohort, ClareV V-gene embeddings are sequence-grounded but not sequence-determined, and their CMV-associated geometric shifts capture information that is largely distinct from classical V-frequency summaries. This is consistent with the classification results showing that ClareV embeddings and V-usage features can be complementary. These embedding-space patterns are intended as hypothesis-generating observations specific to the Emerson2017 cohort, and do not establish a causal or mechanistic explanation for the observed CMV-associated structure.

## 4 Discussion

We introduced ClareV as a contrastive-learning framework that learns data-driven TRBV representations from repertoire context rather than treating V categories as fixed count features. This design preserves cohort-specific immune-background information and links predictive modeling with structural interpretation in a unified pipeline. In the CMV case study, ClareV features consistently improved discrimination over usage-only and simple-pooling baselines across both V-family and V-gene settings. Comparison against non-contrastive learned-bag baselines further supports that this gain comes from the contrastive objective itself rather than from learned bag aggregation alone.

Representing V genes as learned embeddings rather than fixed categorical or count features is useful because it allows inter-gene similarity and cohort-specific representational proximity to be modeled explicitly, while retaining a compact feature space amenable to downstream inspection. The embedding space analyses support this interpretability: ClareV partially preserved sequence-grounded V-gene organization while also capturing repertoire-context structure that was largely distinct from classical V-frequency summaries. Usage-matched analyses further support that ClareV retains repertoire-level signal beyond coarse V-usage aggregation. Because class prevalence can distort threshold-based summaries, we use AUC as the primary metric and treat ACC/F1 as complementary diagnostics, with detailed analyses in **Supplementary Note 1.12** and **Supplementary Figures 6**, **S7**, **S10**, **S11**.

The current scope is constrained by data structure and follow-up availability. This work uses cross sectional repertoires, which are better suited to accumulated immune-history signals than short-term infection dynamics, and currently available longitudinal resources for repeated repertoire follow-up remain limited. A current limitation of ClareV is that V-gene categories must be shared across repertoires for matched V-bag contrastive training, requiring sparse V genes to be filtered before representation learning. To ensure that this preprocessing choice did not artificially weaken V-usage baselines, we additionally trained usage-only classifiers on the full unfiltered V-gene feature space; these models still did not exceed ClareV-based performance (**Supplementary Table 6**). Future versions could relax this fixed-category requirement by using permutation-invariant set encoders over observed V-gene bags, together with masked self-supervised objectives that learn from partially observed V-category sets rather than requiring every V category to be present across all repertoires (51, 52).

A further limitation is that ClareV characterizes only the *β*-chain V gene and does not incorporate HLA information, even though HLA genotype shapes the *αβ* TCR repertoire from the outset, selecting V genes through germline-encoded CDR1 and CDR2 contacts with HLA and thereby skewing the TRBV and TRAV repertoires before any antigen exposure (53, 54). Future repertoire-context representation methods should therefore explicitly encode HLA features, for example by conditioning V-gene embeddings on HLA genotype or by predicting HLA as an auxiliary task (55).

Such embedding-based analyses may be extended to other repertoire cohorts as hypothesis-generating tools for repertoire-context remodeling and candidate biomarker discovery, with their biological relevance verified through future experimental validation, and their clinical relevance tested in longitudinal studies using paired repertoire, treatment, and response data. Methodologically, future extensions could replace the current triplet-loss formulation with more recent self-supervised or multi-view contrastive objectives, especially when larger cross-cohort repertoire resources become available (39, 40). Biologically, the per-clonotype encoder in the current pipeline is TCR2vec, which could be substituted by alternative TCR encoders that exploit structural information (10). Future models could also jointly represent V genes, J genes, and CDR3 sequence context. Prior TCR prediction frameworks often combine these features by concatenation or joint encoding (23, 24), whereas future repertoire-level models could explicitly learn dependencies among them, such as the relationship between V-gene context and the V-proximal region of CDR3.

## Data Availability

The code and trained ClareV models for this study are publicly available at https://github.com/floretli/ClareV. Processed datasets and necessary intermediate results are available at Zenodo (https://zenodo.org/records/15173766). The raw repertoire datasets used in this study are publicly available under the following identifiers: Emerson2017 and Hamm2020 from immuneACCESS/Adaptive Biotechnologies (emerson-2017-natgen and tcrbv4-control, respectively), and Wang2022 from CNCB OMIX (OMIX001106).
