# Supplementary material for: ClareV: a contrastive learning framework for context-aware TRBV representations in TCR repertoires
Source: Front Immunol. 2026 Jul 23;17:1866480. doi: 10.3389/fimmu.2026.1866480 (PMC13443025; doi:10.3389/fimmu.2026.1866480)
Supplement: Supplementary file 1 [file DataSheet1.pdf]

ClareV: Supplementary Materials

1 Supplemental notes

1.1 Repertoire preprocessing truncation and V-bag size justification

This analysis examines two preprocessing choices used before ClareV representation learning: top-10,000 clonotype truncation at the repertoire level and the fixed V-bag size used for frequency-weighted subbag sampling. ClareV preprocessing truncates each repertoire to its top-10,000 clonotypes by frequency before constructing frequency-weighted subbags. To assess the practical impact of this truncation, we examined the raw clone count distributions for 100 randomly sampled Emerson2017 repertoires without any truncation applied. For each repertoire, clonotypes were ranked by count in descending order, and count-vs-rank curves were plotted in log–log scale with a reference line at rank 10,000 to visualize where the truncation threshold falls within the natural frequency distribution (Supplementary Fig. S1).

The analysis reveals a pronounced long-tail distribution: the median rank at which clone count first reaches 1 is 25,092.5 (IQR: 17,606–32,429), placing rank 10,000 solidly within the low-count plateau. In the pooled tail beyond rank 10,000, singleton clonotypes (count = 1) account for 78.1% of entries; clonotypes with count ≤ 2 and ≤ 3 account for 92.3% and 92.6%, respectively. Because ClareV constructs subbags by frequency-weighted sampling ($p_i = w_i / \sum_j w_j$), these ultra-low-count clones receive near-zero sampling probability and are effectively excluded from any subbag even when present. The truncation therefore has negligible practical influence on the learned embeddings.

Separately, we evaluated the expected number of unique clonotypes sampled per subbag as a function of bag size n across all three cohorts (Supplementary Fig. S3A), and the empirical bag diversity (unique clones / n) at the default $n = 50$ per (repertoire, V-family) cell (Supplementary Fig. S3B). Across cohorts, the diversity ratio at $n = 50$ has a median of approximately 0.75–0.85, indicating that the default bag size yields substantially diverse subbags with minimal redundancy, supporting the choice of $n = 50$ as the operating point.

1.2 Triplet margin hyperparameter sensitivity

To assess the sensitivity of ClareV to the triplet loss margin γ, we trained five Emerson2017 V-family extractors with $\gamma \in \{0.05, 0.08, 0.10, 0.15, 0.20\}$, holding

all other hyperparameters fixed (40 training epochs, learning rate 10^{-4}, subbag size 50, 8:1:1 train/validation/test split). For each γ, we embedded a random subset of 200 repertoires and computed (i) UMAP projections of the resulting repertoire-specific V-family bag embeddings, colored by V-family identity and by repertoire identity (Supplementary Fig. S4), and (ii) V-family alignment metrics—NMI, ARI, Purity, and Silhouette coefficient—evaluated by k-means clustering at multiple k (Supplementary Fig. S5).

The UMAP topology is broadly stable across $\gamma = 0.05$–0.10: V-family clusters remain compact and well-separated, and the per-repertoire point distributions are consistent across panels. V-family alignment metrics confirm this range as well-behaved: NMI decreases modestly from 0.883 ($\gamma = 0.05$) to 0.758 ($\gamma = 0.10$), and the Silhouette coefficient remains positive throughout (0.201 to 0.075). Beyond $\gamma = 0.10$, degradation accelerates markedly: at $\gamma = 0.15$, NMI drops to 0.552 and Silhouette to 0.010; at $\gamma = 0.20$, NMI reaches 0.352 and Silhouette becomes negative (-0.019), indicating that embeddings no longer respect V-family structure. We therefore recommend $\gamma \in [0.05, 0.10]$ as the practical operating range for discovery-scale cohorts. For smaller validation cohorts with fewer training triplets, a conservative value of $\gamma = 0.08$ is preferable to reduce the risk of embedding collapse; this choice is consistent with the degradation trend observed on Emerson2017 and was applied to both validation cohorts in this study (Wang2022 n = 123; Hamm2020 n = 147).

1.3 External validation TCR repertoire cohorts

To assess generalizability beyond the Emerson2017 cohort, we additionally evaluated ClareV on two independent PBMC TCR repertoire cohorts at both V-family and V-gene resolution. We restricted the choice to studies in which patient and healthy repertoires were sequenced within the same protocol, so that the two label classes share the same technical pipeline and the model is not driven by batch effects between cohorts.

The Wang2022 cohort (1) contains 123 PBMC repertoires, including 74 gastric cancer repertoires and 49 controls (CNCB OMIX, accession OMIX001106).

The Hamm2020 cohort (2) contains 147 PBMC repertoires, including 59 cancer repertoires and 88 healthy controls (Adaptive Biotech, accession `tcrbv4-control`). Both external cohorts were preprocessed with the same V-bag construction pipeline as the Emerson2017 cohort (see Methods).

Each cohort is evaluated under the same nested 5-fold cross-validation protocol as the Emerson2017 experiments: outer-test repertoires are held out from both extractor training and downstream classifier fitting, so the reported AUC contains no leakage between the contrastive embedding learning and the held-out evaluation. The contrastive extractor is trained with the same triplet loss as in the main experiments,

$$\mathcal{L}_{cl} = \sum_{\text{triplets}} \max\big(-\operatorname{Sim}\big(\Phi(X_{\text{anchor}}), \Phi(X_{\text{pos}})\big) + \operatorname{Sim}\big(\Phi(X_{\text{anchor}}), \Phi(X_{\text{neg}})\big) + \gamma,\ 0\big)\ ,$$

in which the margin γ sets the required separation between matched and unmatched bag pairs. For these smaller cohorts (Wang2022, Hamm2020), a more conservative margin and a shorter training schedule than for Emerson2017 are used to limit overfitting on the smaller triplet pool; the specific per-cohort training hyperparameters are listed in Methods Section 2.4.

1.4 Implementation details for repertoire classifiers

For the repertoire-level classification task, we compared ClareV-based classifiers with baseline models operating on V usage features. The baseline models included Logistic Regression, Random Forest, support vector machines (SVM) with linear and RBF kernels, a one-dimensional convolutional neural network (CNN), and a multilayer perceptron (MLP).

The Logistic Regression baseline used L2 regularization with balanced class weighting ($C = 1.0$). The Random Forest baseline used 100 decision trees with Gini impurity splitting. The SVM baselines were evaluated with both linear and RBF kernels, where the latter used automatic tuning of the γ parameter. The CNN baseline used 8 convolutional filters with kernel size 3, followed by a flattened layer and a sigmoid-activated fully connected layer. The MLP baseline used 128 hidden units with ReLU activation and was trained with early stopping based on an inner validation split (patience of five epochs) within each outer-training fold.

For ClareV-based classifiers, the single-modality classifier was trained with Adam, whereas the RF Fusion model was trained with AdamW. Both used an initial learning rate of 10^{-4}, a batch size of 16, and up to 30 training epochs, with early stopping based on an inner validation split (patience of five epochs). All ClareV and baseline classifiers used identical nested cross-validation splits to ensure matched outer test folds across methods.

The RF Fusion model combines the ClareV embedding with the V-frequency signal at the logit level. A Random Forest classifier trained on V-frequency features produces a per-repertoire positive-class probability p_{RF}, and its logit $\mathrm{logit}(p_{\mathrm{RF}})$ is added to the output of the embedding branch through a learnable scalar. This residual skip lets the model retain the V-frequency baseline when the embedding branch carries limited additional signal. The embedding branch first maps the aggregated ClareV embedding through a bottleneck layer and then combines it with p_{RF} to produce the residual logit on top of the Random Forest skip.

Because the ClareV preprocessing pipeline removes a small number of sparse V-gene categories before representation learning, the main method comparison uses the same retained V-gene feature set for ClareV-based models and V-usage baselines. This matched feature space avoids giving non-contextual repertoire-level classifiers V categories that are unavailable to the ClareV models. As an additional sensitivity analysis, we trained non-contextual repertoire-level classifiers using the full unfiltered V-gene feature space; these results are reported in Supplementary Tab. S6.

1.5 Evaluation metrics for repertoire classification

Here we present the detailed formulas for the performance metrics used to evaluate repertoire classification tasks. The accuracy (ACC) quantifies the proportion of correctly classified samples and is defined as

$$\text{ACC} = \frac{1}{N} \sum_{i=1}^{N} \mathbb{I}(y_i = \hat{y}_i),$$

where N represents the total number of samples, y_i is the true label, $\hat{y}_i$ is the predicted label for the ith sample, and $\mathbb{I}$ is the indicator function that returns 1 when its argument is true and 0 otherwise.

The area under the ROC curve (AUC) is defined by the integral

$$\text{AUC} = \int_0^1 \text{TPR}(\theta)\, d\text{FPR}(\theta),$$

where $\text{TPR}(\theta)$ and $\text{FPR}(\theta)$ are the true positive rate and false positive rate, respectively, as functions of the classification threshold θ.

The F1 score, which balances precision and recall, is computed as

$$\text{F1 score} = \frac{2 \cdot \text{Precision} \cdot \text{Recall}}{\text{Precision} + \text{Recall}}.$$

In this work, these metrics are computed on the outer test folds in nested 5-fold cross-validation and then averaged across folds. For methods requiring model selection (e.g., early stopping), an inner validation split is used only within the corresponding outer-training data and is never used for final metric reporting.

1.6 Implementation details for non-contrastive learned V-bag baselines

To provide a learned-bag control without a contrastive objective, we implemented two non-contrastive baselines: a supervised V-bag encoder and an Attention MIL V-bag encoder. Both baselines use the same V-bag inputs and the same predefined nested 5-fold repertoire splits as the main ClareV classification experiments.

The supervised V-bag encoder uses the same bag-encoder architecture as ClareV but removes the separately pretrained contrastive extractor. For a repertoire represented as $X \in \mathbb{R}^{K \times n \times 120}$, where K is the number of retained V categories and n is the subbag size, each clone embedding $\mathbf{x}_{k,i}$ in V bag k is transformed by the ClareV-style instance encoder,

$$\mathbf{h}_{k,i} = \text{ReLU}(\text{LayerNorm}(W_1 \mathbf{x}_{k,i}))\,,$$

then summarized by mean pooling and a second projection,

$$\mathbf{z}_k = W_2 \left(\frac{1}{n} \sum_{i=1}^{n} \mathbf{h}_{k,i} \right).$$

The resulting per-V representations $\{\mathbf{z}_k\}_{k=1}^{K}$ are flattened and passed to a repertoire-level classifier. The encoder and classifier are optimized jointly with cross-entropy on labelled repertoires, so this baseline tests whether supervised end-to-end learning from the same V bags can replace ClareV's triplet-based contrastive pretraining.

The Attention MIL V-bag encoder extends the supervised version by replacing uniform mean pooling over clonotypes within each V bag with a learned gated-attention aggregator. After the same instance-level projection, attention scores are computed within each V bag as

$$a_{k,i} = \frac{\exp\big(\mathbf{w}^\top [\tanh(V\mathbf{h}_{k,i}) \odot \sigma(U\mathbf{h}_{k,i})]\big)}{\sum_{j=1}^{n} \exp(\mathbf{w}^\top [\tanh(V\mathbf{h}_{k,j}) \odot \sigma(U\mathbf{h}_{k,j})])}, \qquad \mathbf{z}_k = W_o \sum_{i=1}^{n} a_{k,i}\mathbf{h}_{k,i}.$$

This model therefore learns which clonotypes within a V-specific bag should contribute most strongly to the repertoire-level prediction, while still using only supervised repertoire labels and no contrastive triplets.

The resulting per-method AUC, ACC, and F1 summaries are reported in Supplementary Tabs. S1 and S2.

1.7 Metrics for quantitatively assessing the alignment between clustering and IMGT V gene classification

We compare the clustering results of V gene embedding representations against the ground truth V gene labels derived from the IMGT annotation. To quantify the degree of alignment with the original V gene classification in the embedding space, we compute four indicators over different numbers of clusters (k) using k-means clustering.

1. **1-NMI:**

   Normalized Mutual Information (NMI) evaluates how much information the clustering assignments share with the original V gene labels. Let C represent the clustering outcome and L the set of V gene labels from IMGT. NMI is defined as

   $$\text{NMI}(C, L) = \frac{2\, I(C, L)}{H(C) + H(L)},$$

   where the mutual information $I(C, L)$ is computed by

   $$I(C, L) = \sum_{c \in C} \sum_{l \in L} P(c, l) \log \left(\frac{P(c, l)}{P(c)P(l)} \right).$$

   Here, $P(c)$ and $P(l)$ denote the probabilities of a sample being in cluster c or having a V gene label l, respectively, and $P(c, l)$ represents their joint probability. In this setting, a higher NMI indicates better alignment between the clusters and the original V gene labels. To express misalignment, we define:

   $$\text{1-NMI} = 1 - \frac{2\, I(C, L)}{H(C) + H(L)}.$$

Thus, higher 1-NMI values reflect lower alignment with the original V gene classification.

2. **1-ARI:**

   The Adjusted Rand Index (ARI) assesses the similarity between the clustering result and the IMGT V gene labels, correcting for chance groupings. Let n_{ij} be the number of samples in cluster j with V gene label i, $a_i = \sum_j n_{ij}$ be the total number of samples with label i, $b_j = \sum_i n_{ij}$ be the number of samples in cluster j, and N be the total sample size. The ARI is given by

$$\text{ARI} = \frac{\displaystyle\sum_i \sum_j \binom{n_{ij}}{2} - \frac{\displaystyle\sum_i \binom{a_i}{2} \sum_j \binom{b_j}{2}}{\binom{N}{2}}}{\displaystyle\frac{1}{2}\left[\sum_i \binom{a_i}{2} + \sum_j \binom{b_j}{2}\right] - \frac{\displaystyle\sum_i \binom{a_i}{2} \sum_j \binom{b_j}{2}}{\binom{N}{2}}}.$$

   A higher ARI indicates greater alignment between the clustering and the original V gene labels. To represent misalignment, we define:

$$\text{1-ARI} = 1 - \text{ARI}.$$

   Accordingly, higher 1-ARI values indicate lower alignment with the original V gene classification.

3. **1-Purity:**

   Purity measures the extent to which each cluster is dominated by a single V gene label (3). For each cluster j, if $\max_i n_{ij}$ is the maximum number of samples sharing the same V gene label, then the purity is calculated as

$$\text{Purity} = \frac{1}{N} \sum_j \max_i n_{ij}.$$

   High purity indicates that clusters are well-aligned with the original V gene categories. To express misalignment, we define:

$$\text{1-Purity} = 1 - \left(\frac{1}{N} \sum_j \max_i n_{ij} \right).$$

   Higher values of 1-Purity thus signify lower alignment with the original V gene classification.

4. **Cluster Entropy Measure (4):**

Entropy quantifies the uncertainty in the distribution of V gene labels within each cluster. For a given cluster j containing n_j samples, the entropy is computed as

$$H(j) = -\sum_i \left(\frac{n_{ij}}{n_j}\right) \log \left(\frac{n_{ij}}{n_j}\right).$$

The overall cluster entropy is then defined as the weighted average:

$$H_{\text{total}} = \frac{1}{N} \sum_j n_j \, H(j).$$

A higher H_{total} indicates a more uniform distribution of V gene labels within clusters, reflecting a lower alignment with the original IMGT classification.

1.8 Quantitative evaluation of V family structure

Quantitative evaluation of the V family structure was performed by computing silhouette coefficients(5) for the embeddings. For each embedding, the average intra-family distance (a) was calculated as the mean distance to all other embeddings within the same V family, while the minimum inter-family distance (b) was determined as the smallest average distance to embeddings from a different V family. The silhouette coefficient (s) for each embedding was then computed using the formula

$$s = \frac{b - a}{\max(a, b)},$$

where a value close to 1 indicates that an embedding is well-clustered within its V family and clearly separated from other families, and values near 0 or negative suggest overlapping clusters. The overall mean silhouette coefficient across all embeddings quantitatively reflects the extent to which the ClareV-learned embeddings align with the IMGT V family organization.

1.9 Detailed methodology for hierarchical clustering analysis

In this analysis, we applied a well-trained TCR bag extractor to all repertoires in the Emerson2017 cohort to generate corresponding V gene matrices. A mean V embedding was computed by averaging the V gene embeddings across all repertoires. Hierarchical clustering was then performed on this aggregated embedding matrix using cosine distance as the similarity measure to construct a dendrogram that reflects the embedding-space relationships among V genes.

Let $\mathbf{e}_i^{\text{vgene}}$ and $\mathbf{e}_j^{\text{vgene}}$ denote the embeddings for the ith and jth V gene, respectively. The cosine distance was computed as:

$$d_{\text{vgene}}(i, j) = 1 - \frac{\mathbf{e}_i^{\text{vgene}} \cdot \mathbf{e}_j^{\text{vgene}}}{\|\mathbf{e}_i^{\text{vgene}}\| \, \|\mathbf{e}_j^{\text{vgene}}\|},$$

which quantifies the similarity between V gene embeddings based on their angular difference. The resulting distance matrix served as the basis for the hierarchical clustering procedure.

IMGT-defined V family annotations were then appended to the hierarchical clustering tree for clarity and reference.

1.10 V-embedding geometry analyses

1.10.1 Embedding inputs and V-gene centroids

These analyses were performed on the Emerson2017 dataset at V-gene resolution, using the same trained ClareV extractor used for the V-gene visualization experiments. For each repertoire, each retained V-gene group was encoded by the extractor. To reduce stochastic variation from subbag sampling, embeddings were averaged over five independent forward passes for sequence-related analyses and ten forward passes for CMV+/− shift analyses.

For each V gene, the ClareV centroid was computed by averaging its repertoire-specific embeddings across Emerson2017 repertoires. As a sequence-only baseline, the corresponding TCR2vec centroid was computed by averaging raw TCR2vec clonotype embeddings assigned to the same V gene across the same repertoires. This matched setup keeps the repertoire set, clonotype set, and V-gene grouping fixed, so differences between centroid geometries primarily reflect the ClareV projection and contrastive training rather than changes in input composition.

1.10.2 Sequence-reference comparison

Human TRBV amino-acid sequences were taken from the IMGT reference (6), retaining IMGT-style gap characters to preserve sequence alignment. Pairwise sequence identity was computed in a gap-aware manner: only positions where both V genes carry a residue were counted, and identity was defined as the fraction of matching residues among those shared positions.

For each embedding family (ClareV and TCR2vec baseline), we computed the pairwise V-V cosine distance matrix $D_{ij} = 1 - \cos(\mathbf{c}_i, \mathbf{c}_j)$. We then compared this embedding-distance matrix with the sequence-distance matrix $1 - \text{identity}_{ij}$ using a Mantel test (7), with Spearman rank correlation as the test statistic. The permutation reference distribution was generated from $B = 10{,}000$ independent row-and-column permutations of one distance matrix, preserving matrix symmetry. The reported P_{perm} is the right-tailed empirical probability $P_{\text{perm}} = (1 + |\{k : \rho_k \geq \rho_{\text{obs}}\}|)/(1 + B)$, where ρ_k is the rank correlation under the k-th permutation.

1.10.3 Within-family distance analysis

To test whether V genes from the same IMGT family are closer in embedding space than genes from different families, we compared within-family and between-family pairwise cosine distances. In the retained V-gene set, seven IMGT families contain two or more V genes (TRBV4, TRBV5, TRBV6, TRBV7, TRBV10,

TRBV11, TRBV12), yielding 25 within-family and 570 between-family V-V pairs. The two pair populations were compared with a one-sided Mann-Whitney U test (alternative hypothesis: within-family distance < between-family distance). The same procedure was applied separately to the ClareV and TCR2vec baseline distance matrices.

1.11 Per-V CMV-associated embedding shifts

1.11.1 Embedding-shift statistic

To examine whether individual V genes show CMV-associated changes in ClareV space, we partitioned the Emerson2017 repertoires by CMV status and computed, for each V gene, the centroid of its ClareV embeddings in the CMV-positive and CMV-negative groups. The per-V shift magnitude was defined as the Euclidean distance between these two centroids. The corresponding shift direction was defined as the normalized vector from the CMV-negative centroid to the CMV-positive centroid.

1.11.2 Permutation assessment

We assessed statistical evidence for the embedding shifts by permuting CMV labels across repertoires and recomputing the per-V centroid distances under each permutation. This label-permutation null preserves the repertoire structure and V-gene embedding distribution while removing CMV-status association. Per-V empirical P-values were computed as right-tailed permutation probabilities with standard plus-one correction. The same permutation framework was also used for the directional-consistency statistic described below.

1.11.3 Comparison with classical V-usage signal

To compare the embedding-based signal with conventional repertoire summaries, we independently computed a classical per-V usage statistic. For each V gene, normalized V-usage frequencies were compared between CMV-positive and CMV-negative repertoires using a two-sample Welch t-test, and the absolute t-statistic was used as the V-usage ranking score. The embedding-shift ranking and V-usage ranking were then compared using Spearman rank correlation with a two-tailed P-value.

1.11.4 Directional-consistency control

Finally, we tested whether the per-V shift directions were jointly structured rather than independent V-specific noise. For each V gene, the CMV-positive to CMV-negative shift was converted to a unit vector, and the mean resultant length R across V genes was used as the directional-consistency statistic. Larger R indicates that V-specific shifts point in more similar directions. Significance was assessed with the same CMV-label permutation null used above.

Observed values were $R = 0.67$ for ClareV ($P_{\text{perm}} < 10^{-3}$; permutation-null median 0.26, 95th percentile 0.37) and $R = 0.87$ for the TCR2vec baseline ($P_{\text{perm}} < 10^{-3}$; permutation-null median 0.56). Because directional consistency is at least as strong in raw TCR2vec averages as in the contrastive embedding, we do not attribute this directional structure specifically to the contrastive layer. Instead, it is reported as evidence that per-V CMV-associated shifts are jointly non-random, consistent with known systemic effects of CMV infection on the TCR repertoire (8, 9, 10).

1.12 Diagnostic interpretation of ACC/F1 and evidence beyond V-usage summaries

Because the Emerson2017 cohort has a positive-majority class balance (420/760 CMV-positive), ACC/F1 should be interpreted together with class-specific diagnostics. In pooled outer-test evaluation at the V-family protocol (Supplementary Fig. S10–S11), two usage-only baselines illustrate this issue clearly: baseline MLP reached F1 = 0.714 with specificity = 0.065, and baseline CNN reached F1 = 0.712 with specificity = 0.000. Their corresponding AUC values remained comparatively low (0.641 and 0.527, respectively), indicating weak discrimination despite superficially high F1.

We use AUC as the primary discrimination metric and report ACC/F1 as secondary summaries (Supplementary Tabs. S1 and S2).

To evaluate whether ClareV captures signal beyond coarse V-usage aggregation, we additionally analyzed usage-matched gene-level comparisons against the strongest usage-only baseline (Random Forest). ClareV yielded more complementary correct calls (128 ClareV-correct/RF-wrong vs 106 RF-correct/ClareV-wrong). In nearest-neighbor usage-matched pairs, pairwise ranking accuracy was 0.6214 for Random Forest and 0.6738 for ClareV. In the high-similarity subset (cosine ≥ 0.95), pairwise ranking accuracy was 0.5964 vs 0.6641, with an absolute gain of 0.0677 (95% CI: 0.0080 to 0.1300). Representative matched cases are shown in Supplementary Fig. S6–S7.

2 Supplemental figure

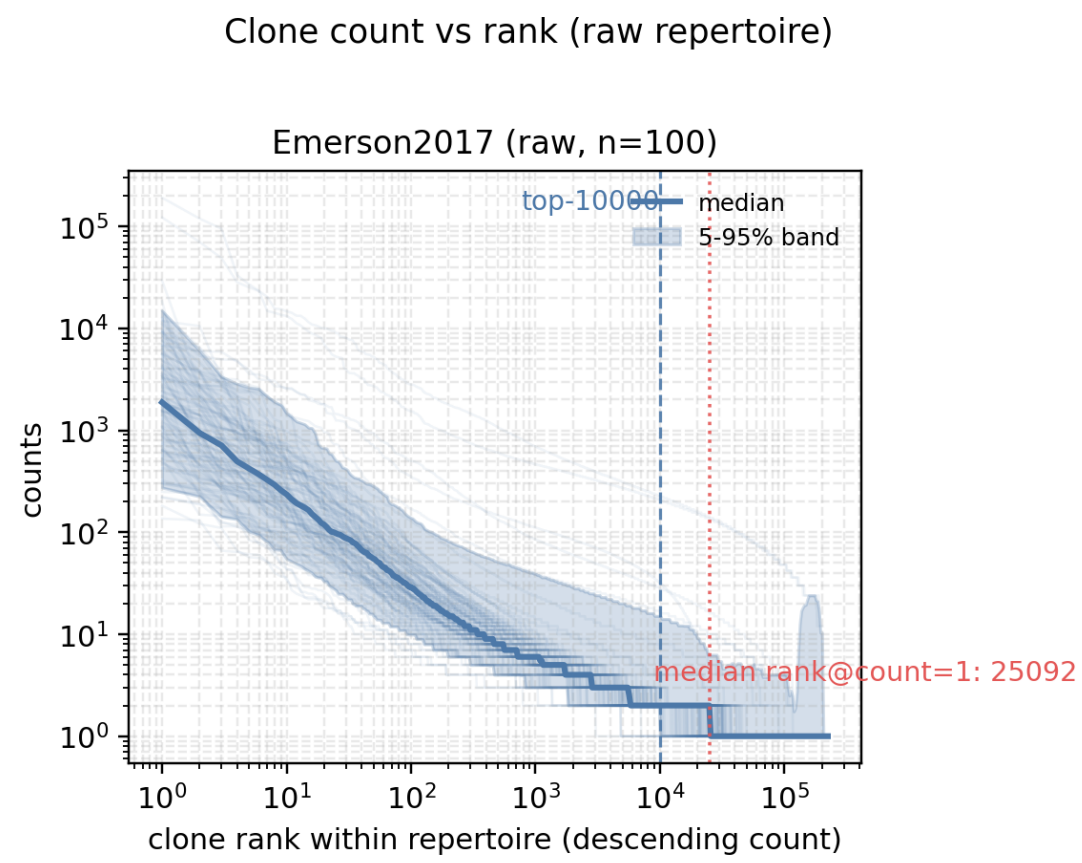

Figure S1: **Clone-count distribution relative to the top-10,000 truncation threshold.** Clone count vs. rank in log–log scale for 100 randomly sampled Emerson2017 repertoires before truncation. The shaded band shows the 5th to 95th percentile range; the solid line is the median. The dashed vertical line marks rank 10,000 (the truncation threshold), and the dashed horizontal line marks the median rank at which clone count first reaches 1 (rank 25,093).

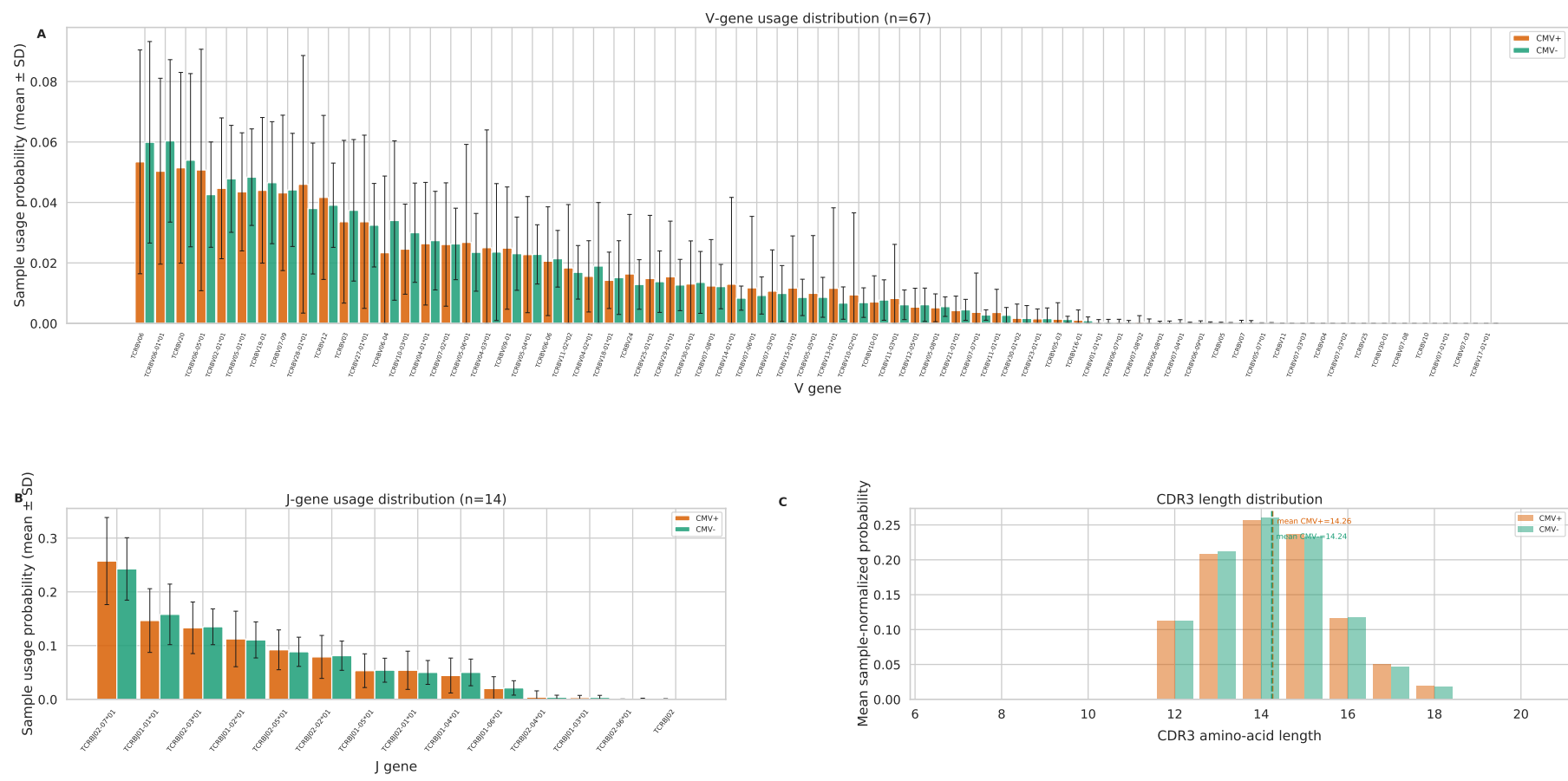

Figure S2: **Distribution summaries of V-gene usage, J-gene usage, and CDR3 length in the Emerson2017 cohort.** (**A**) Sample-level V-gene usage probabilities for all non-zero V genes, shown as group means with standard deviation T-bars across repertoires. (**B**) Sample-level J-gene usage probabilities for all non-zero J genes, shown as group means with standard deviation T-bars across repertoires. (**C**) CDR3 amino-acid length distribution shown as group-mean sample-normalized probabilities, with dashed vertical lines marking group mean CDR3 lengths.

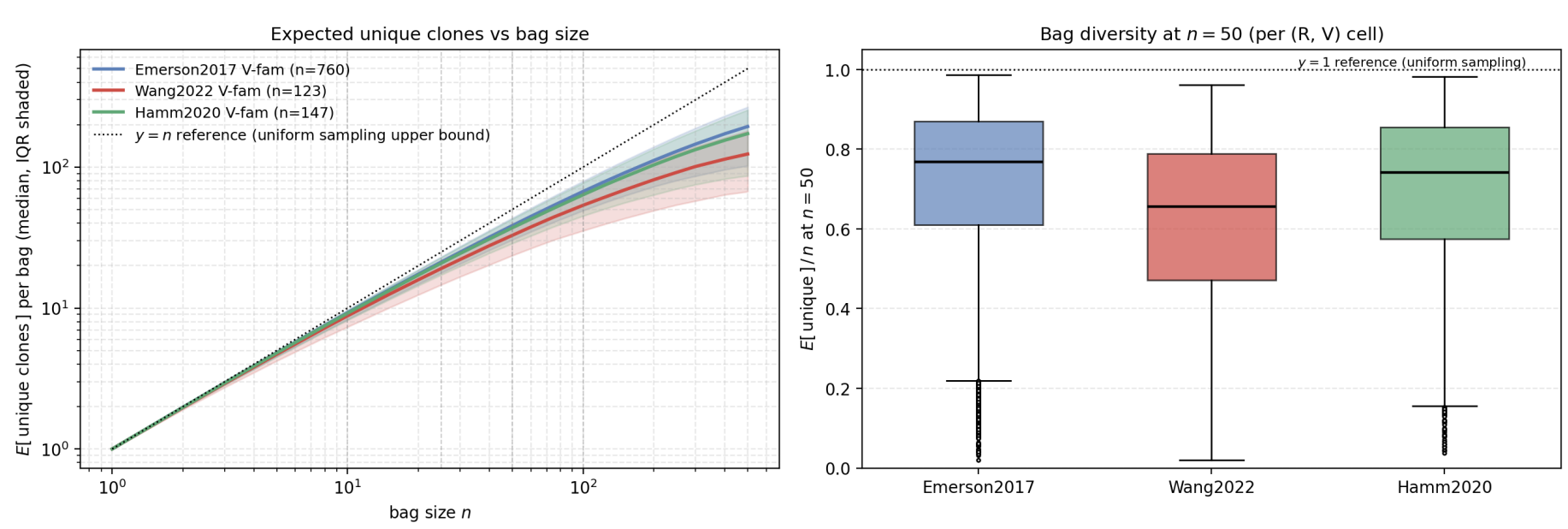

Figure S3: **V-bag sampling diversity as a function of bag size.** (**A**) Expected number of unique clones per subbag as a function of bag size n for all three cohorts (median with IQR); the diagonal $y = n$ line is the theoretical uniform-sampling upper bound. (**B**) Empirical bag diversity (unique clones / n) at the default bag size $n = 50$, per (repertoire, V-family) cell, shown as boxplots across cohorts.

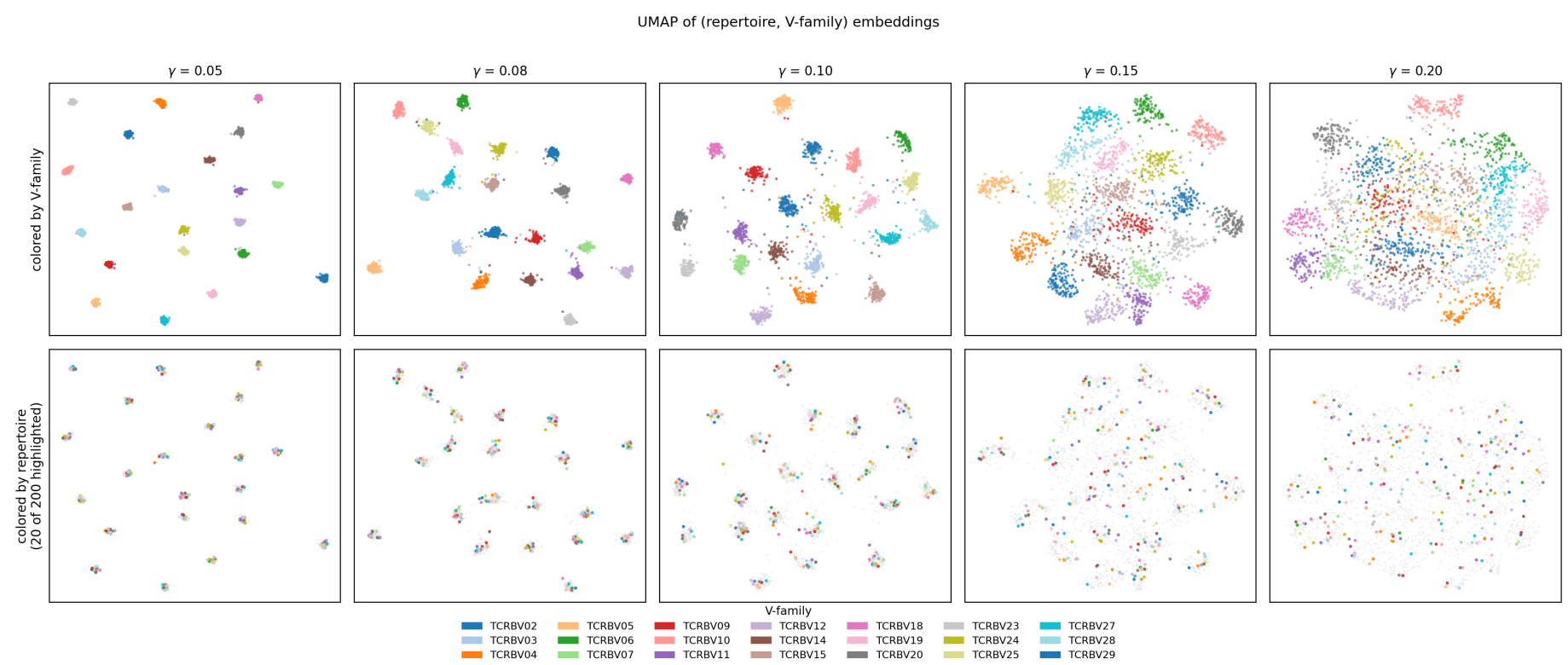

Figure S4: **UMAP of repertoire-specific V-family bag embeddings across triplet margin values.** Each column corresponds to one margin value ($\gamma \in \{0.05, 0.08, 0.10, 0.15, 0.20\}$). **Top row:** points colored by V-family identity (legend below). **Bottom row:** points colored by repertoire identity, with 20 randomly selected repertoires highlighted and the remainder shown in gray. At $\gamma = 0.05$–0.10, V-family clusters are compact and well-separated; beyond $\gamma = 0.10$ the embedding progressively loses V-family structure, with visible collapse at $\gamma = 0.20$. Embeddings were computed on 200 randomly subsampled Emerson2017 repertoires; UMAP was applied to up to 2,500 points per panel.

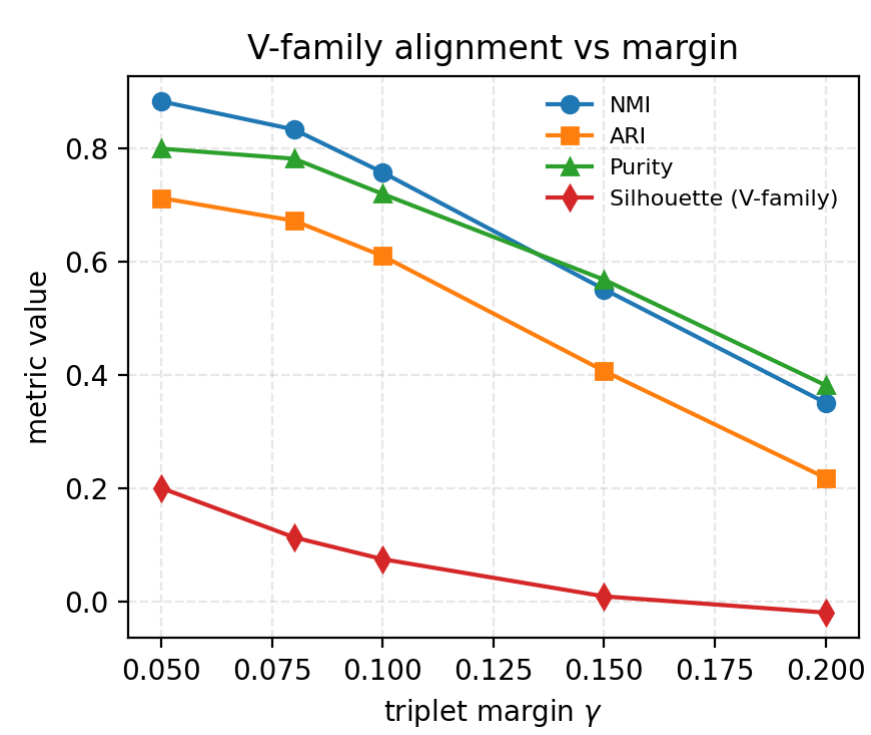

Figure S5: **V-family alignment metrics of ClareV embeddings as a function of triplet margin γ.** NMI, ARI, Purity, and Silhouette coefficient are computed by k-means clustering of repertoire-specific V-family bag embeddings against ground-truth V-family labels (averaged over $k \in \{10, 21, 50\}$). All four metrics decrease monotonically with γ. The sharpest decline occurs between $\gamma = 0.10$ and $\gamma = 0.15$, and the Silhouette coefficient turns negative at $\gamma = 0.20$, indicating that embeddings at that margin no longer recover V-family structure.

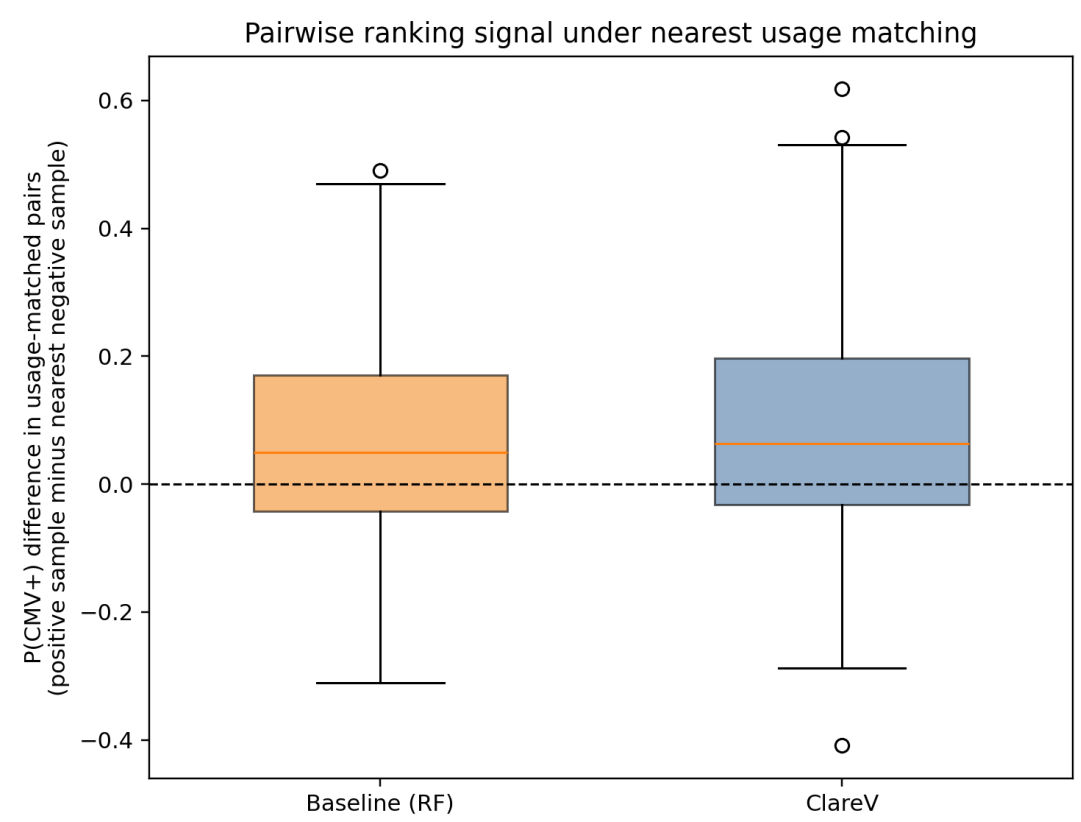

Figure S6: **Usage-matched Emerson2017 CMV probability separation (gene-level; ClareV vs Random Forest usage baseline).** Each CMV+ repertoire is paired with its nearest CMV- repertoire in normalized V-gene usage space (cosine similarity). The plotted value is $\Delta P(\text{CMV+})$ (positive sample minus matched negative sample). ClareV exhibits a stronger positive shift than the usage-only Random Forest baseline, consistent with improved pairwise ranking under controlled usage similarity.

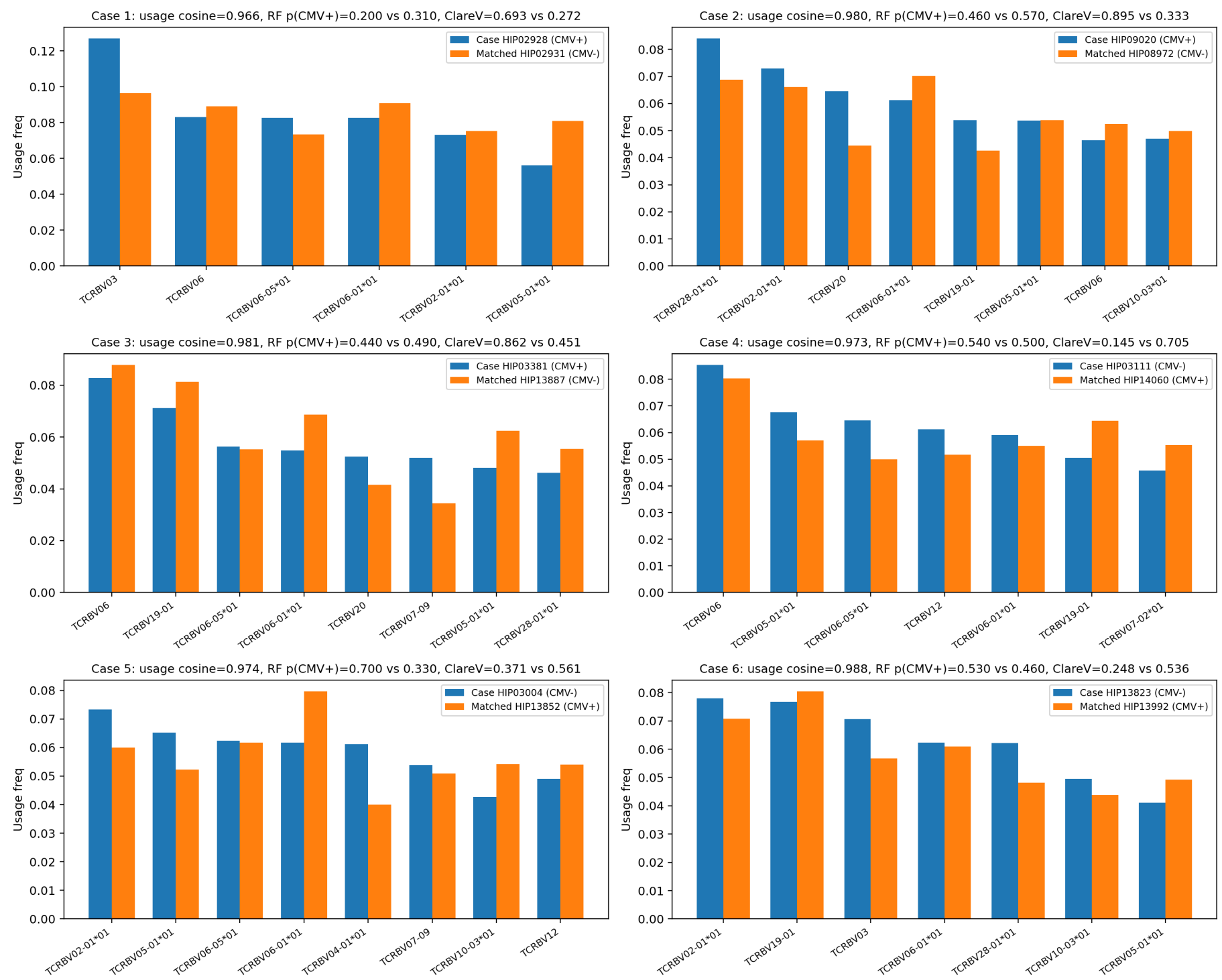

Figure S7: **Multiple representative usage-matched case profiles (gene-level; ClareV-correct and Random Forest-wrong cases).** Each panel shows one representative case and its nearest opposite-label match in V-gene usage space, including top V-gene usage frequencies and model probabilities. Across multiple examples, high usage similarity does not prevent ClareV from producing clearer label-consistent probability separation than the usage-only baseline, supporting the interpretation that ClareV captures additional repertoire-level information beyond aggregated V-gene usage.

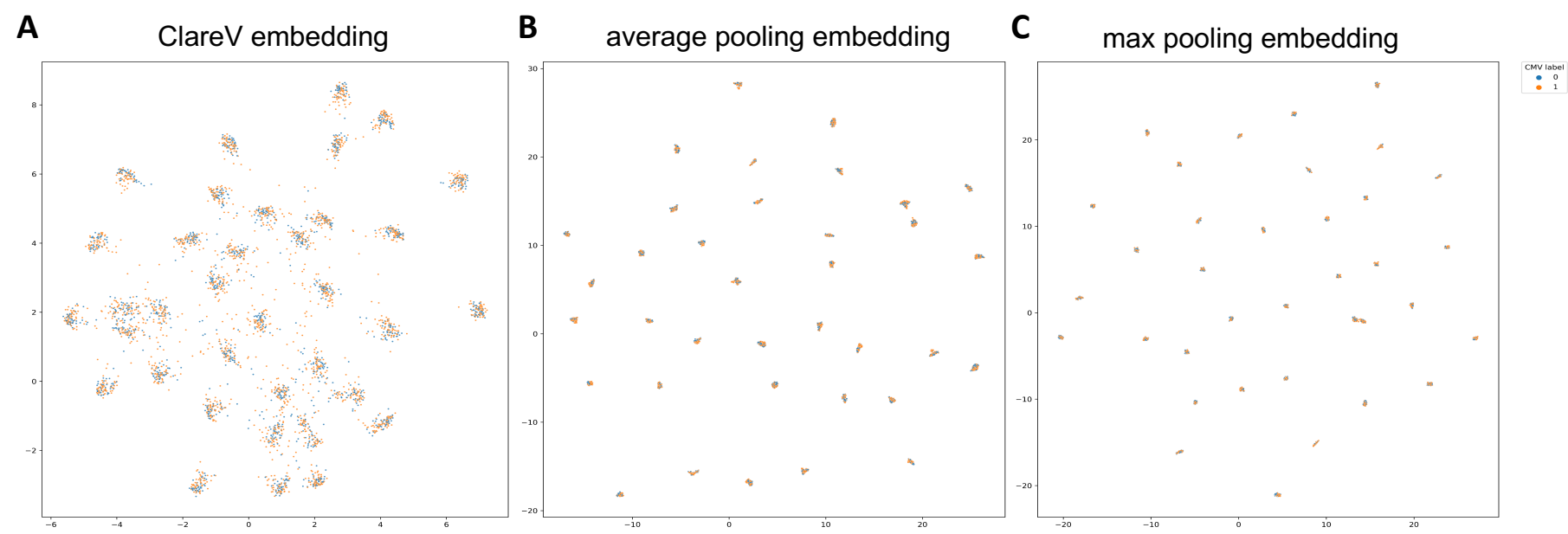

Figure S8: **UMAP visualization of V gene embeddings colored by CMV status.** UMAP projections for 100 randomly selected Emerson2017 repertoires, with points colored by CMV status (orange: CMV-positive; blue: CMV-negative). Panels (**A**) to (**C**) correspond to ClareV, average pooling, and max pooling embeddings, respectively.

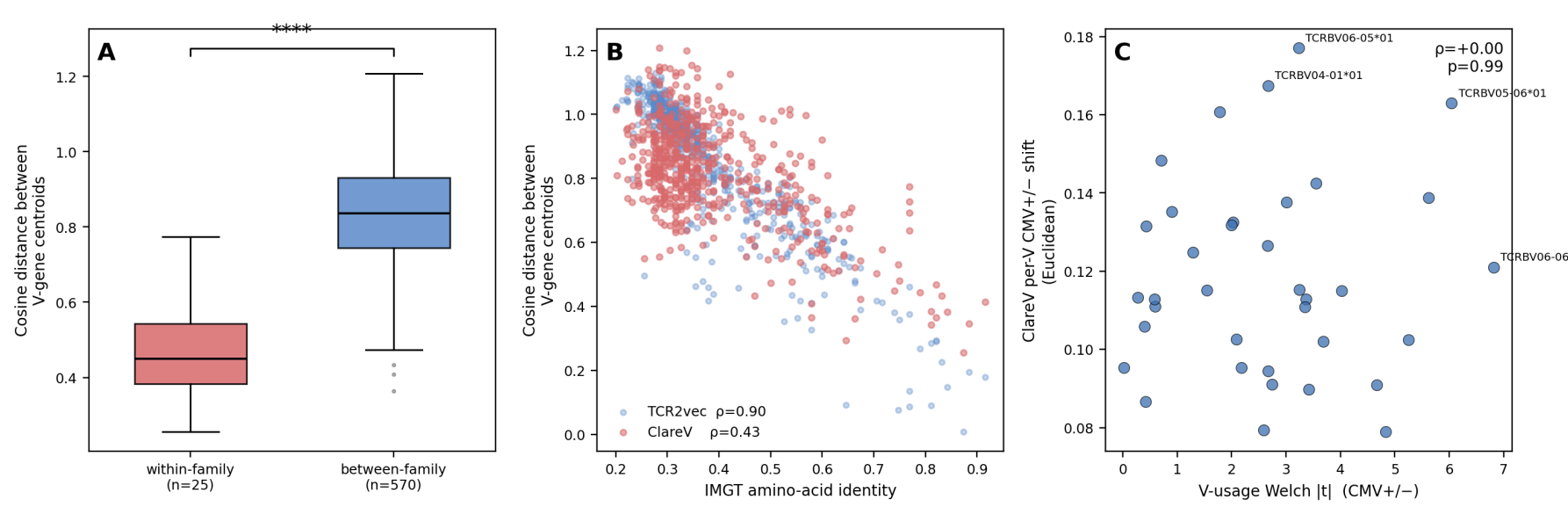

Figure S9: **V-embedding interpretability summary on Emerson2017.** (**A**) Within-family vs. between-family pairwise cosine distance in the ClareV embedding ($N = 35$ V genes; 25 within-family and 570 between-family pairs). Bracket: one-sided Mann-Whitney; **** denotes $P < 10^{-4}$. (**B**) Pairwise V-V cosine distance vs. IMGT amino-acid identity. Each dot is a V-V pair; blue = TCR2vec baseline, red = ClareV. Mantel Spearman correlations are reported in the legend ($P_{\text{perm}} < 10^{-3}$ for both). (**C**) Per-V CMV+/− responsiveness in the two views. Horizontal axis: classical V-usage Welch $|t|$ on per-repertoire frequencies; vertical axis: ClareV per-V CMV+/− centroid Euclidean shift. Spearman ρ and P are reported in the corner. Labels mark the top two V genes by each axis.

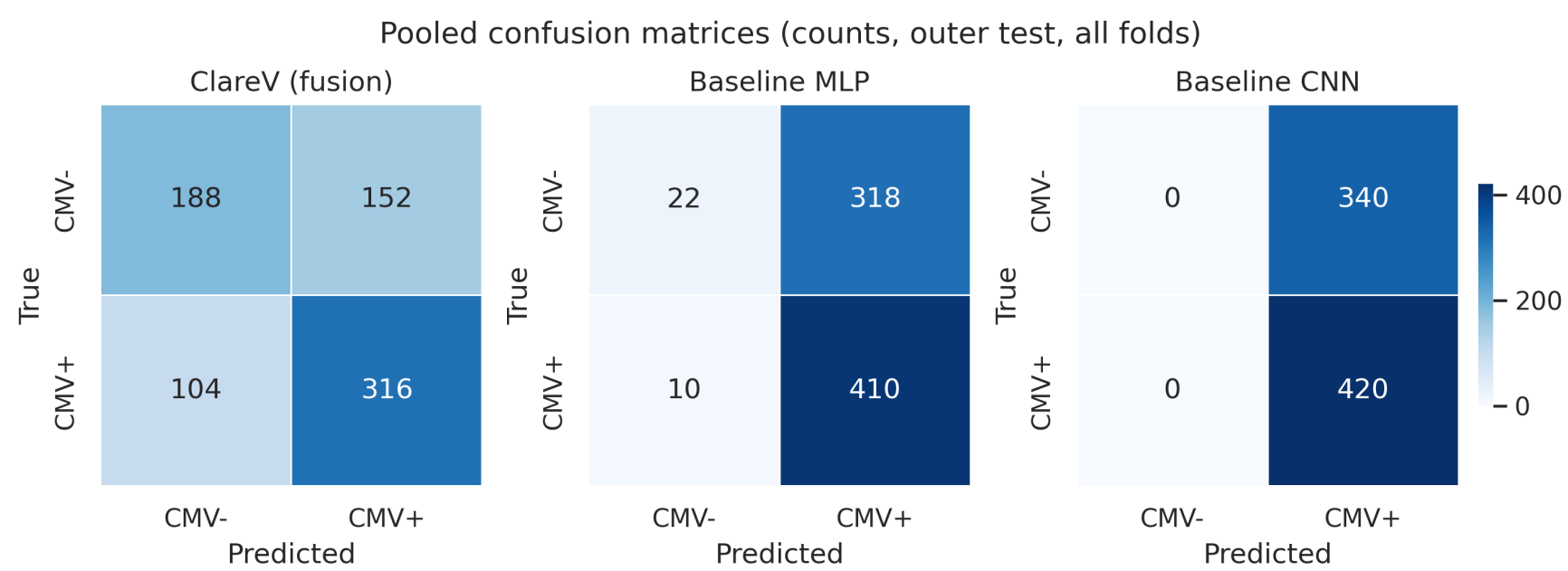

Figure S10: **Pooled confusion matrices for Emerson2017 CMV classification at the V-family protocol.** Confusion matrices were computed on pooled Emerson2017 outer-test predictions under identical labels and split protocol. Rows denote true labels and columns denote predicted labels. The three panels share the same color scale, enabling direct comparison of prediction tendencies. Compared with ClareV fusion, some usage-only baselines show stronger positive-class bias with reduced true-negative recovery.

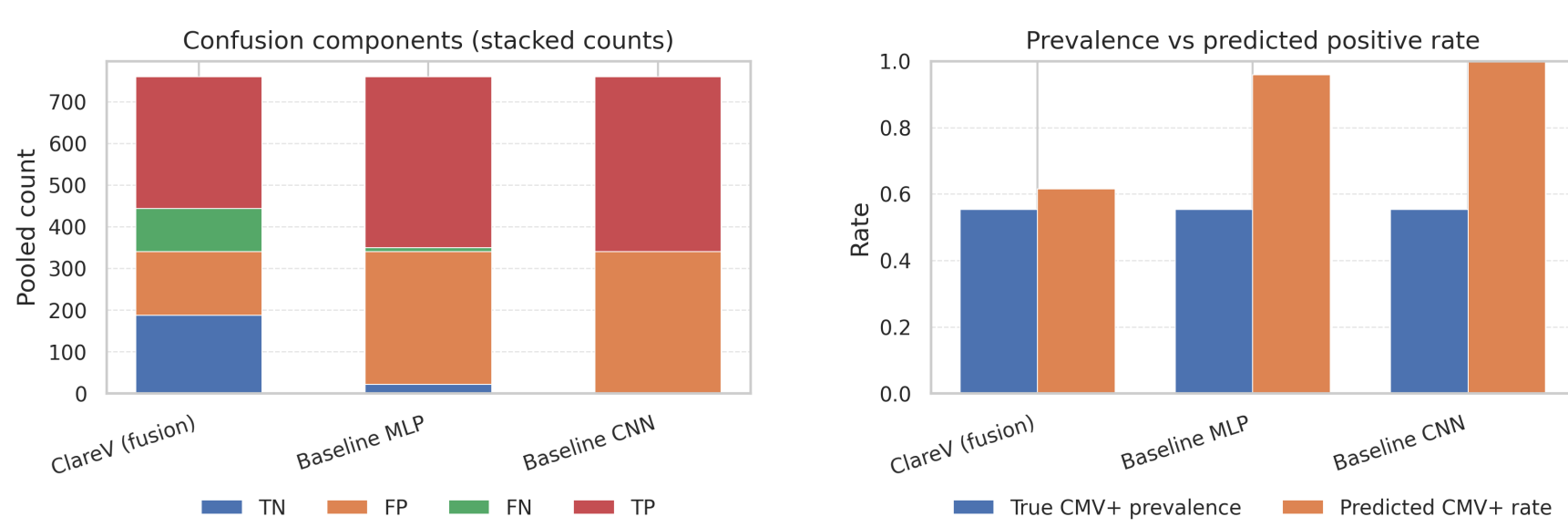

Figure S11: **Confusion components and predicted-positive rate diagnostics for Emerson2017 CMV classification.** Left: stacked counts of TN/FP/FN/TP from pooled outer-test predictions. Right: comparison between the true CMV+ prevalence and each method's predicted-positive rate. The diagnostics show that high F1 can coincide with heavily positive-biased predictions, motivating AUC-centered comparison for discrimination quality.

3 Supplemental table

Table S1: **Emerson2017 V-family classification performance.** Nested 5-fold test performance; values are mean ± SD across outer folds, with 95% bootstrap CIs for AUC.

Feature Type	Method	AUC	AUC 95% CI	ACC	F1
ClareV-based	Fusion model	**0.776 ± 0.028**	(0.742–0.809)	0.711 ± 0.034	0.726 ± 0.026
	ClareV embedding only	0.755 ± 0.022	(0.721–0.788)	0.634 ± 0.070	0.717 ± 0.022
Non-CL V-bag	Attention MIL V-bag	0.689 ± 0.070	(0.652–0.725)	0.572 ± 0.068	0.716 ± 0.034
	Supervised V-bag	0.663 ± 0.107	(0.626–0.700)	0.554 ± 0.035	0.712 ± 0.028
V-usage only	Random Forest	0.669 ± 0.035	(0.630–0.707)	0.618 ± 0.044	0.665 ± 0.041
	Logistic Regression	0.674 ± 0.016	(0.635–0.710)	0.612 ± 0.036	0.640 ± 0.050
	SVM (linear)	0.679 ± 0.012	(0.640–0.717)	0.637 ± 0.039	0.686 ± 0.042
	SVM (RBF)	0.678 ± 0.013	(0.638–0.715)	0.605 ± 0.021	0.671 ± 0.027
	MLP	0.672 ± 0.014	(0.631–0.709)	0.564 ± 0.018	0.717 ± 0.021
	CNN	0.527 ± 0.115	(0.487–0.568)	0.553 ± 0.033	0.711 ± 0.028

Note: The Fusion model combines ClareV embeddings with V-usage features (see Methods). Non-CL denotes non-contrastive learned V-bag baselines. Bold indicates the best AUC.

Table S2: **Emerson2017 V-gene classification performance.** Nested 5-fold test performance; values are mean ± SD across outer folds, with 95% bootstrap CIs for AUC.

Feature Type	Method	AUC	AUC 95% CI	ACC	F1
ClareV-based	Fusion model	**0.782 ± 0.034**	(0.749–0.813)	0.704 ± 0.040	0.713 ± 0.047
	ClareV embedding only	0.756 ± 0.024	(0.722–0.788)	0.672 ± 0.018	0.700 ± 0.017
Non-CL V-bag	Attention MIL V-bag	0.687 ± 0.048	(0.650–0.725)	0.583 ± 0.054	0.697 ± 0.053
	Supervised V-bag	0.693 ± 0.036	(0.655–0.730)	0.553 ± 0.033	0.711 ± 0.027
V-usage only	Random Forest	0.690 ± 0.029	(0.652–0.727)	0.625 ± 0.056	0.673 ± 0.052
	Logistic Regression	0.662 ± 0.005	(0.622–0.700)	0.596 ± 0.020	0.616 ± 0.036
	SVM (linear)	0.674 ± 0.016	(0.635–0.712)	0.608 ± 0.032	0.669 ± 0.036
	SVM (RBF)	0.676 ± 0.022	(0.637–0.714)	0.611 ± 0.024	0.680 ± 0.022
	MLP	0.624 ± 0.091	(0.585–0.664)	0.539 ± 0.051	0.572 ± 0.321
	CNN	0.484 ± 0.043	(0.440–0.526)	0.553 ± 0.033	0.711 ± 0.028

Note: The Fusion model combines ClareV embeddings with V-usage features (see Methods). Non-CL denotes non-contrastive learned V-bag baselines. Bold indicates the best AUC.

Table S3: **Independent validation cohort classification performance.** Nested 5-fold AUC summaries for Wang2022 and Hamm2020; values are mean ± SD across outer folds, with 95% bootstrap CIs.

**Cohort**	**Resolution**	**Feature Type**	**Method**	**AUC**	**AUC 95% CI**
Wang2022	V-family	ClareV-based	Fusion model	$\mathbf{0.899 \pm 0.041}$	(0.832–0.956)
Wang2022	V-family	ClareV-based	ClareV embedding only	0.791 ± 0.160	(0.702–0.872)
Wang2022	V-family	Non-CL V-bag	Attention MIL V-bag	0.665 ± 0.123	(0.560–0.766)
Wang2022	V-family	Non-CL V-bag	Supervised V-bag	0.593 ± 0.090	(0.484–0.699)
Wang2022	V-family	V-usage only	Random Forest	0.847 ± 0.042	(0.769–0.913)
Wang2022	V-family	V-usage only	Logistic Regression	0.649 ± 0.141	(0.548–0.750)
Wang2022	V-family	V-usage only	SVM (Linear)	0.790 ± 0.041	(0.697–0.875)
Wang2022	V-family	V-usage only	SVM (RBF)	0.796 ± 0.058	(0.701–0.880)
Wang2022	V-family	V-usage only	MLP	0.705 ± 0.187	(0.606–0.800)
Wang2022	V-family	V-usage only	CNN	0.486 ± 0.099	(0.379–0.592)
Wang2022	V-gene	ClareV-based	Fusion model	$\mathbf{0.930 \pm 0.053}$	(0.876–0.970)
Wang2022	V-gene	ClareV-based	ClareV embedding only	0.861 ± 0.065	(0.784–0.925)
Wang2022	V-gene	Non-CL V-bag	Attention MIL V-bag	0.764 ± 0.127	(0.670–0.847)
Wang2022	V-gene	Non-CL V-bag	Supervised V-bag	0.698 ± 0.099	(0.600–0.794)
Wang2022	V-gene	V-usage only	Random Forest	0.845 ± 0.114	(0.769–0.910)
Wang2022	V-gene	V-usage only	Logistic Regression	0.700 ± 0.076	(0.598–0.795)
Wang2022	V-gene	V-usage only	SVM (Linear)	0.787 ± 0.068	(0.697–0.867)
Wang2022	V-gene	V-usage only	SVM (RBF)	0.830 ± 0.083	(0.751–0.901)
Wang2022	V-gene	V-usage only	MLP	0.431 ± 0.203	(0.333–0.534)
Wang2022	V-gene	V-usage only	CNN	0.517 ± 0.192	(0.417–0.621)
Hamm2020	V-family	ClareV-based	Fusion model	$\mathbf{0.819 \pm 0.104}$	(0.748–0.884)
Hamm2020	V-family	ClareV-based	ClareV embedding only	0.736 ± 0.081	(0.649–0.816)
Hamm2020	V-family	Non-CL V-bag	Attention MIL V-bag	0.681 ± 0.106	(0.581–0.773)
Hamm2020	V-family	Non-CL V-bag	Supervised V-bag	0.677 ± 0.063	(0.579–0.771)
Hamm2020	V-family	V-usage only	Random Forest	0.770 ± 0.106	(0.689–0.847)
Hamm2020	V-family	V-usage only	Logistic Regression	0.728 ± 0.097	(0.636–0.809)
Hamm2020	V-family	V-usage only	SVM (Linear)	0.723 ± 0.072	(0.629–0.808)
Hamm2020	V-family	V-usage only	SVM (RBF)	0.713 ± 0.095	(0.623–0.798)
Hamm2020	V-family	V-usage only	MLP	0.594 ± 0.087	(0.497–0.691)
Hamm2020	V-family	V-usage only	CNN	0.426 ± 0.268	(0.338–0.514)
Hamm2020	V-gene	ClareV-based	Fusion model	$\mathbf{0.807 \pm 0.105}$	(0.730–0.875)
Hamm2020	V-gene	ClareV-based	ClareV embedding only	0.766 ± 0.108	(0.686–0.846)

Table S3: Independent validation cohort classification performance (continued).

Cohort	Resolution	Feature Type	Method	AUC	AUC 95% CI
Hamm2020	V-gene	Non-CL V-bag	Attention MIL V-bag	0.621 ± 0.139	(0.524–0.713)
Hamm2020	V-gene	Non-CL V-bag	Supervised V-bag	0.645 ± 0.126	(0.551–0.738)
Hamm2020	V-gene	V-usage only	Random Forest	0.796 ± 0.095	(0.721–0.866)
Hamm2020	V-gene	V-usage only	Logistic Regression	0.749 ± 0.128	(0.660–0.831)
Hamm2020	V-gene	V-usage only	SVM (Linear)	0.751 ± 0.105	(0.663–0.834)
Hamm2020	V-gene	V-usage only	SVM (RBF)	0.747 ± 0.123	(0.660–0.827)
Hamm2020	V-gene	V-usage only	MLP	0.633 ± 0.177	(0.535–0.725)
Hamm2020	V-gene	V-usage only	CNN	0.441 ± 0.107	(0.341–0.539)

Note: Bold values indicate the best AUC within each cohort/resolution block.

Table S4: **Paired ΔAUC between ClareV RF Fusion and reference baselines.** For each cohort/resolution combination, ΔAUC is the fold-mean AUC difference on matched outer-fold predictions. The 95% CI and one-sided $P(\Delta \leq 0)$ are from a fold-stratified paired bootstrap ($B = 5000$) using identical resampled patient indices for both methods within each fold. Asterisks indicate a 95% CI that excludes 0.

Cohort / Resolution	Reference baseline	ΔAUC	95% CI	$P(\Delta \leq 0)$	Sig.
Emerson2017 V-family	Random Forest	+0.107	(+0.077, +0.138)	< 0.001	*
Emerson2017 V-family	Attention MIL V-bag	+0.087	(+0.050, +0.123)	< 0.001	*
Emerson2017 V-family	Supervised V-bag	+0.113	(+0.074, +0.153)	< 0.001	*
Emerson2017 V-gene	Random Forest	+0.092	(+0.061, +0.122)	< 0.001	*
Emerson2017 V-gene	Attention MIL V-bag	+0.095	(+0.060, +0.132)	< 0.001	*
Emerson2017 V-gene	Supervised V-bag	+0.088	(+0.053, +0.124)	< 0.001	*
Wang2022 V-family	Random Forest	+0.052	(−0.013, +0.120)	0.059	
Wang2022 V-family	Attention MIL V-bag	+0.234	(+0.129, +0.342)	< 0.001	*
Wang2022 V-family	Supervised V-bag	+0.306	(+0.192, +0.422)	< 0.001	*
Wang2022 V-gene	Random Forest	+0.085	(+0.026, +0.150)	0.003	*
Wang2022 V-gene	Attention MIL V-bag	+0.166	(+0.075, +0.266)	< 0.001	*
Wang2022 V-gene	Supervised V-bag	+0.232	(+0.127, +0.338)	< 0.001	*
Hamm2020 V-family	Random Forest	+0.049	(+0.006, +0.097)	0.014	*
Hamm2020 V-family	Attention MIL V-bag	+0.139	(+0.042, +0.238)	0.002	*
Hamm2020 V-family	Supervised V-bag	+0.142	(+0.042, +0.245)	0.002	*
Hamm2020 V-gene	Random Forest	+0.011	(−0.026, +0.051)	0.290	
Hamm2020 V-gene	Attention MIL V-bag	+0.186	(+0.074, +0.299)	< 0.001	*
Hamm2020 V-gene	Supervised V-bag	+0.162	(+0.054, +0.267)	0.001	*

Table S5: **Silhouette coefficients for the V gene clusters derived from ClareV embeddings.** Negative coefficients indicate that embeddings are, on average, closer to neighboring clusters than samples within their own cluster.

Cluster	Silhouette Coefficient
Average	0.04224
TCRBV02	0.1412
TCRBV03	0.1424
TCRBV04	0.0375
TCRBV05	-0.0977
TCRBV06	-0.0148
TCRBV07	0.0118
TCRBV09	0.1469
TCRBV10	0.0279
TCRBV11	0.0598
TCRBV12	0.0465
TCRBV14	0.1008
TCRBV15	0.1324
TCRBV18	0.1851
TCRBV19	0.1311
TCRBV20	0.2607
TCRBV23	-0.0171
TCRBV24	-0.0075
TCRBV25	0.1337
TCRBV27	0.1314
TCRBV28	0.1545
TCRBV29	0.0405

Table S6: **Usage-only baseline performance with unfiltered V-gene feature space.** Nested 5-fold cross-validation with the same predefined data split as the main experiments; values are reported as mean ± standard deviation over outer test folds. ClareV models are intentionally excluded here.

Resolution	Method	ACC	AUC	F1
V-family	SVM (RBF)	0.6105 ± 0.0281	0.6809 ± 0.0115	0.6819 ± 0.0348
V-family	SVM (Linear)	0.6289 ± 0.0281	0.6788 ± 0.0150	0.6890 ± 0.0320
V-family	Logistic Regression	0.6145 ± 0.0368	0.6736 ± 0.0180	0.6457 ± 0.0451
V-family	Random Forest	0.5947 ± 0.0394	0.6549 ± 0.0375	0.6610 ± 0.0363
V-gene	Random Forest	0.6237 ± 0.0525	0.7072 ± 0.0275	0.6841 ± 0.0455
V-gene	SVM (RBF)	0.6079 ± 0.0120	0.6773 ± 0.0101	0.6834 ± 0.0164
V-gene	SVM (Linear)	0.6158 ± 0.0423	0.6736 ± 0.0161	0.6837 ± 0.0459
V-gene	Logistic Regression	0.6053 ± 0.0168	0.6635 ± 0.0041	0.6285 ± 0.0304

References

1 . Wang M, Gao P, Ren L, Duan J, Yang S, Wang H, et al. Profiling the peripheral blood t cell receptor repertoires of gastric cancer patients. *Frontiers in Immunology* **13** (2022) 848113.

2 . [Dataset] Hamm D. immunoseq hstcrb-v4 control data (2020). doi:10.21417/ADPT2020V4CD.

3 . Valkiers S, Van Houcke M, Laukens K, Meysman P. Clustcr: a python interface for rapid clustering of large sets of cdr3 sequences with unknown antigen specificity. *Bioinformatics* **37** (2021) 4865–4867. doi:10.1093/bioinformatics/btab446.

4 . Rosenberg A, Hirschberg J. V-measure: A conditional entropy-based external cluster evaluation measure. *Proceedings of the 2007 joint conference on empirical methods in natural language processing and computational natural language learning (EMNLP-CoNLL)* (2007), 410–420.

5 . Aranganayagi S, Thangavel K. Clustering categorical data using silhouette coefficient as a relocating measure. *International conference on computational intelligence and multimedia applications (ICCIMA 2007)* (IEEE) (2007), vol. 2, 13–17. doi:10.1109/ICCIMA.2007.328.

6 . Lefranc MP, Pommié C, Ruiz M, Giudicelli V, Foulquier E, Truong L, et al. Imgt unique numbering for immunoglobulin and t cell receptor variable domains and ig superfamily v-like domains. *Developmental & Comparative Immunology* **27** (2003) 55–77. doi:10.1016/S0145-305X(02)00039-3.

7 . Mantel N. The detection of disease clustering and a generalized regression approach. *Cancer Research* **27** (1967) 209–220.

8 . Sylwester AW, Mitchell BL, Edgar JB, Taormina C, Pelte C, Ruchti F, et al. Broadly targeted human cytomegalovirus-specific cd4+ and cd8+ t cells dominate the memory compartments of exposed subjects. *Journal of Experimental Medicine* **202** (2005) 673–685. doi:10.1084/jem.20050882.

9 . Britanova OV, Putintseva EV, Shugay M, Merzlyak EM, Turchaninova MA, Staroverov DB, et al. Age-related decrease in tcr repertoire diversity measured with deep and normalized sequence profiling. *The Journal of Immunology* **192** (2014) 2689–2698. doi:10.4049/jimmunol.1302064.

10 . Emerson RO, DeWitt WS, Vignali M, Gravley J, Hu JK, Osborne EJ, et al. Immunosequencing identifies signatures of cytomegalovirus exposure history and hla-mediated effects on the t cell repertoire. *Nature genetics* **49** (2017) 659–665. doi:10.1038/ng.3822.
